# Photoredox-Catalyzed Decarboxylative Cross-Coupling Reaction to Synthesis Unsymmetrical Diarylmethanes

**DOI:** 10.3390/molecules29092156

**Published:** 2024-05-06

**Authors:** Guozhe Guo, Yuquan Zhang, Yanchun Li, Zhijun Li

**Affiliations:** Gansu Key Laboratory of Efficient Utilization of Oil and Gas Resources, College of Petroleum and Chemical Engineering, Longdong University, Qingyang 745000, China

**Keywords:** decarboxylative, cross coupling, unsymmetrical diarylmethanes, photoredox catalyzed

## Abstract

The photoredox-catalyzed decarboxylative cross-coupling reaction of aryl acetic acids and aryl nitriles has been achieved under an argon atmosphere in high yields. This method provides a fast way to obtain prevalent aryl acetic acids from an abundant natural source. A tentative radical mechanism has been proposed.

## 1. Introduction

**Figure 1:**
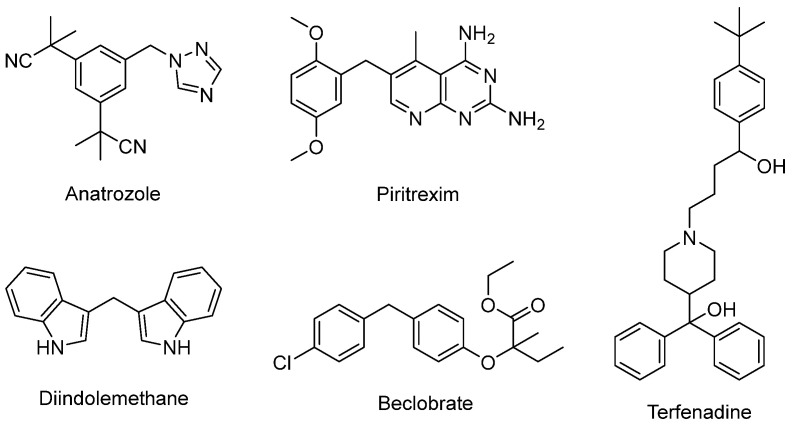
The represent drugs containing diarylmethane scaffolds.

Diarylmethane motifs play a prominent role in the natural, bioactive and pharmaceutical product industries [1,2]. Some examples of anticancer agents include anastrozole [3], pitracoxin [4] and diindolemethane [5] (Figure 1). Anastrozole and letrozole are known to have pharmacological activity against breast cancer. Pharmacological experiments have shown that piritrexim has a significant inhibitory effect on dihydrofolate reductase of leukemia cells and is mainly used in the treatment of malignant tumors. Diindolemethane is present in cruciferous plants and also exhibits anticancer effects. Beclobrate [6] is a potent drug used to lower lipids and is about 20 times more effective than clofibrate. It also has some inhibition activity on platelet aggregation. Terfenadine is a second-generation antihistamine that is more selective to H1 receptors than the first generation and less irritating to the central nervous system [7]. It has been reported that some molecules containing diarylmethane skeleton may have a certain inhibitory effect on COVID-19 [8]. Carboxylic acids are widely employed as raw materials in organic synthesis because of their features of low toxicity, high stability and rich reserves. 3-indoleacetic acid (IAA) was detected by Salkowski in 1885 [9], who demonstrated that it can not only regulate the growth of plants but can also facilitate other aspects of plant development. In addition, the IAA industry is currently maintaining a stable market growth [10]. Therefore, the transformation of IAA and synthesis of diarylmethanes are highly appealing in organic chemistry due to their significant importance.

**Scheme 1:**
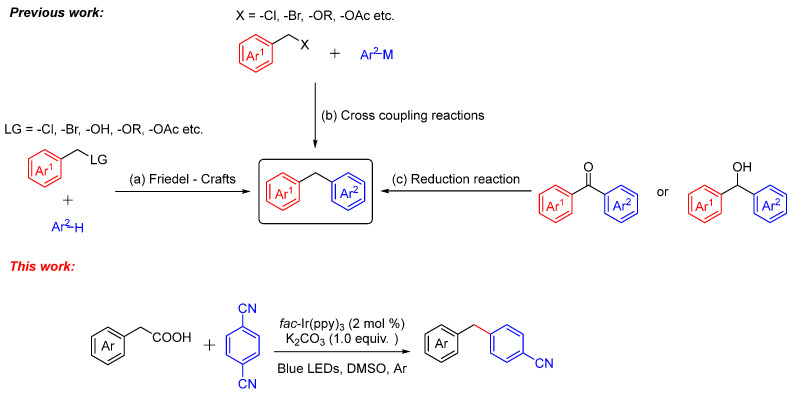
The conventional methods toward synthesis of diarylmethanes.

There are many ways to synthesize diarylmethane [11,12,13,14], and the conventional methods include (Scheme 1) (a) functionalization of arenes by means of a Friedel–Crafts reaction [15,16,17,18], (b) cross-coupling reaction [19,20,21,22,23,24,25,26], and (c) reduction reaction [27,28]. Among them, the typical approaches demand a high temperature and high pressure, which are generally achieved using highly demanded equipment. Recently, direct decarboxylative cross-coupling reactions have attracted researchers’ attention. In 2018, Lundgren and co-workers [29] reported the Cu-catalyzed decarboxylative cross-coupling reaction of aryl acetic acids and boronic esters to diarylmethanes. In 2021, our group [30] reported the decarboxylative cross-coupling reaction of NHPI with indoles to deliver a broad scope of diarylmethanes. Furthermore, photo-catalyzed chemistry takes over a major area in organic synthesis on account of its environmentally friendly. MacMillan [31,32,33,34] and other research groups [35,36,37,38] have explored lots of novel works on photochemical decarboxylation. Aryl nitriles were converted to their radical anion via SET reduction and underwent radical addition followed by decyanation [39,40,41]. With great efforts, we report the photoredox-catalyzed decarboxylative cross-coupling reaction of aryl acetic acids and aryl nitriles under an argon atmosphere in high yields.

## 2. Results

Initially, our investigations focused on the blue LED irradiation of 3-indoleacetic acid (IAA) **1a** and 1,4-dicyanobenzene (1,4-DCB) **2a** in DMSO under argon atmosphere using 2 mol% of *fac*-Ir(ppy)_3_ in the presence of K_2_CO_3_ at room temperature. To our delight, the diarylmethane **3a** was given in 82% isolated yield (Table 1, entry 1). No reaction took place when the reaction was performed without LED irradiation or without *fac*-Ir(ppy)_3_, resulting in the recovery of substrates **1a** and **2a** (Table 1, entries 2–3). When *fac*-Ir(ppy)_3_ as the photosensitizer was replaced by [Ir(dtbbpy)(ppy)_2_]PF_6_, the yield dropped to 53% (Table 1, entry 4). Furthermore, nearly no product was detected when the other photosensitizers including organic photocatalysts eosin Y and rose bengal were employed (Table 1, entries 5–7). The yield of the decarboxylation cross-coupling reaction dropped to 30% in the absence of a base (Table 1, entry 8). This reaction could also proceed when using other bases, such as K_3_PO_4_, NaOAc and CsF, but the yields decreased to some extent (Table 1, entries 9–11). In addition, DMSO was more optimal than other solvents for the transformation, (Table 1, entries 12–14). while the reaction did not occur when DCM was used as the solvent (Table 1, entry 12). The desired product was given with good yield in MeCN and DMA (Table 1, entries 13–14). Finally, we found that only trace amounts of diarylmethane were obtained when the reaction was performed under an air atmosphere (Table 1, entry 15). This result implies a radical involved mechanism in the reaction because of the biradical feature of the O_2_ molecule.

With the above optimal conditions in hand, we examined the scope of aryl acetic acids with 1,4-DCB to decarboxylative cross-coupling reaction. As shown in Table 2, all aryl acetic acid derivatives **1a**–**1h** reacted smoothly with **2a** to obtain the unsymmetrical diarylmethanes **3a**–**3h** in good to excellent yields. First, alkyl and methoxy substituted heterocyclic acids could provide the corresponding products in yields of 73% (**3b**), 76% (**3c**) and 75% (**3d**), respectively. Moreover, phenyl acetic acid derivatives can also deliver this desired transformation (**3e**–**3g**). Homoveratric acid was coupled to 1,4-DCB to furnish product **3f** in 83% yield. It is worth noting that the medicine molecule Naproxen was tolerated with the mild reaction conditions to obtain target product **3h** in moderate yields, which is the property of non-steroidal anti-inflammatory group agents. This implies the synthetic potential of our approaches to medicinal chemistry.

Next, a range of cyanoaromatics and cyano-heteroaromatics were capable of participating decarboxylative cross-coupling reaction with excellent yields (**4a**–**4f**, 71–80%) in Table 3. 2-methylterephthalonitrile was successfully converted into the corresponding product in 80% yield (**4a**). In addition, isonicotinonitrile was tolerated under the standard conditions (**4b**). Finally, it is worth noting that isonicotinonitrile with halogen substituents were all compatible reaction partners (**4c**–**4e**).

**Scheme 2:**
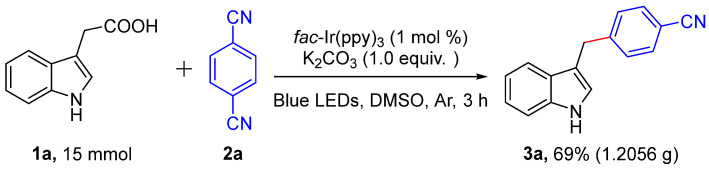
Gram-scale synthesis.

In addition, the practicality of this decarboxylative cross-coupling reaction was further proved by a gram-scale reaction (Scheme 2). When the reaction was carried out under photoredox-catalyzed method, the objected unsymmetrical diarylmethanes **3a** could be gained in high isolated yield (69%).

**Scheme 3:**
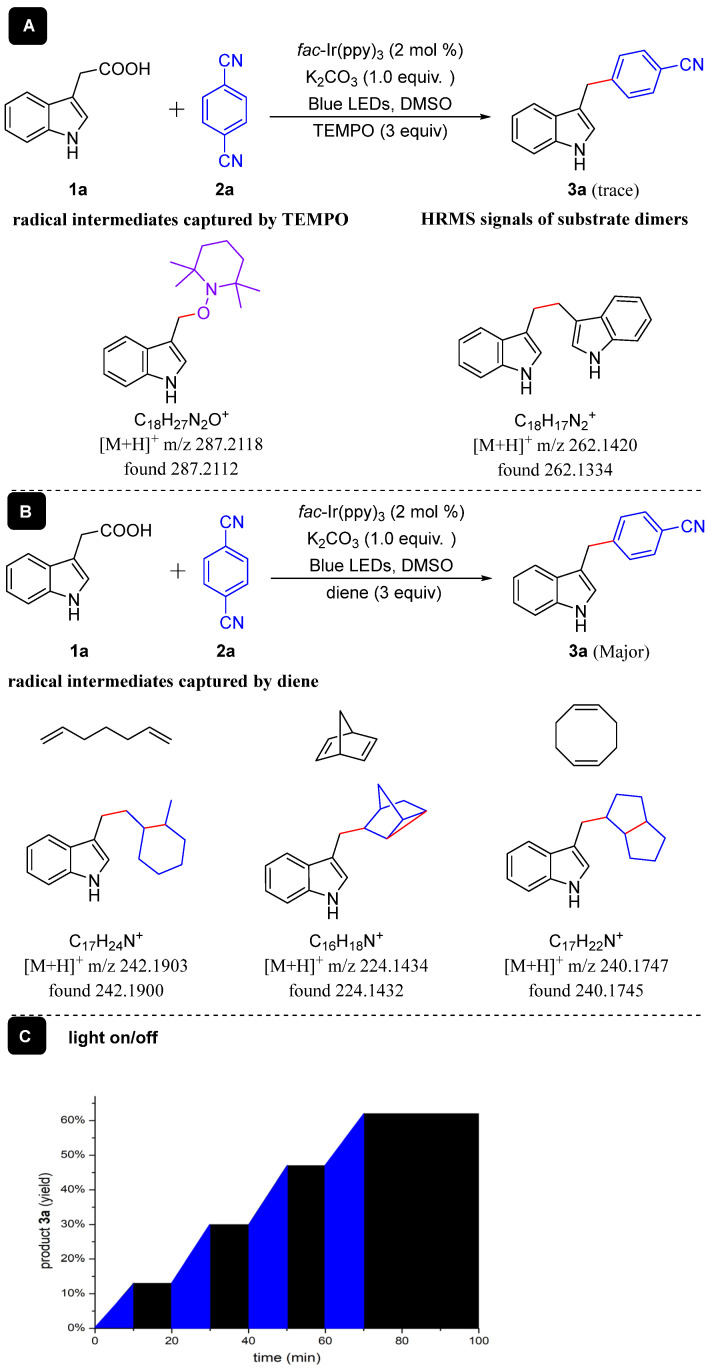
Control experiments.

A series of control experiments were achieved to gain more information about the decarboxylative cross-coupling reaction mechanism between aryl acetic acids and aryl nitriles (Scheme 3). Initially, the reaction of 3-indoleacetic acid (**1a**) with 1,4-DCB (**2a**) was conducted in the presence of radical scavenger TEMPO (2,2,6,6-tetramethylpiperidine) under standard reaction conditions (Scheme 3A), and only a trace amount of compound **3a** was perceived, and TEMPO-trapping compounds could be discerned by HRMS (Scheme 3A), and homocoupling dimer could be detected by HRMS too (Scheme 3A). Then, the radical intermediate in the reaction was compromised by some cyclizations [42] (Scheme 3B). Dienes caused addition/cyclization compatible with intramolecular radical addition based upon HRMS analysis. Finally, the light on/off experiment (Scheme 3C) also verifies that the reaction involves a radical process [43]. These control experiments reveal that this reaction involved radical intermediates.

**Scheme 4:**
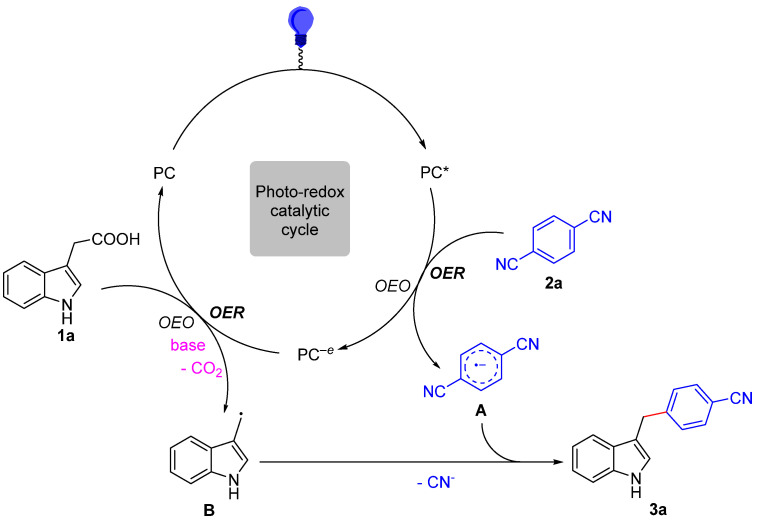
Proposed mechanism.

Guided by the above experimental results and previous literature [44,45,46,47,48,49], a plausible mechanism is presented in Scheme 4 for a photoredox-catalyzed decarboxylative cross-coupling reaction. In the first place, the irradiation of photocatalyst PC [Ir(ppy)_3_] with visible light produces a photoexcited state PC*[*Ir(ppy)_3_], which is a strong reductant (E_1/2_^red^ [Ir^IV^/*Ir^III^] = −1.73 V vs. SCE) [50]. We assumed that the reduction of 1,4-DCB (E_1/2_^red^ = −1.61 V vs. SCE) [51] by single electron transfer to form the corresponding radical anion intermediate **A** and oxidized photocatalyst PC^−e^ [Ir^IV^(ppy)_3_] would be easy. Then, the PC^−e^ [Ir^IV^(ppy)_3_] is a strong oxidant (E_1/2_^red^ = +0.77 V vs. SCE) and could undergo a single electron transfer with 3-indoleacetic acid substrate [52] to deliver the radical intermediate **B** with the elimination of CO_2_, together with regenerating PC [Ir^III^(ppy)_3_] and finishing the photoredox cycle. Finally, the radical–radical coupling of intermediate **A** with **B** and then aromatization with loss of CN^−^ would produce the diarylmethane.

## 3. Materials and Methods

### 3.1. General Information

All materials, reagents and solvents were purchased from commercial suppliers and were used without further purification. Analytical TLC was performed with silica gel GF254 plates, and the products were visualized by UV detection. Flash chromatography was carried out using silica gel 200–300. The ^1^H NMR (400 or 600 MHz) and ^13^C NMR (151 MHz) spectra were measured with CDCl_3_ as solvent. All chemical shifts (δ) are reported in ppm and coupling constants (*J*) in Hz. High-resolution mass spectra (HR-MS) were recorded under electrospray ionization (ESI) conditions.

### 3.2. General Procedure for Light-Promoted Decarboxylative Cross-Coupling Reaction between Aryl Acetic Acids and 1,4-Dicyanobenzene

Aryl acetic acids (**1**, 0.2 mmol), 1,4-dicyanobenzene (**2a**, 0.1 mmol), K_2_CO_3_ (1 equiv.) and *fac*-Ir(ppy)_3_ (2 mol%) were successively added to a dried reaction tube (10 mL) with a magnetic stirring bar. Air was then withdrawn and backfilled with argon 3 times. Subsequently, degassed DMSO (1 mL) was injected into the tube by syringe. Then, the resulting reaction mixture was performed at room temperature under blue LED (6 W) irradiation for 3–24 h. The reaction progress was monitored by TLC. After the reactions were completed, the reaction mixture was diluted with water (10 mL) and washed with EA (3 × 10 mL). The combined organic layers were dried over anhydrous Na_2_SO_4_ and concentrated under reduced pressure. The residue was purified by column chromatography to afford the desired compounds **3** (ethylacetate/n-Hexane = 1:8 to 1:100).

*4-((1H-indol-3-yl)methyl)benzonitrile (**3a**):* The desired pure product was obtained in 82% yield as a white solid. ^1^H NMR (400 MHz, CDCl_3_) δ 8.08 (s, 1H), 7.60–7.52 (m, 2H), 7.47–7.32 (m, 4H), 7.25–7.16 (m, 1H), 7.14–7.04 (m, 1H), 6.98 (d, *J* = 2.3 Hz, 1H), 4.18 (s, 2H). ^13^C NMR (151 MHz, CDCl_3_) δ 147.0, 136. 5, 132.2, 129.3, 127.1, 122.6, 122.3, 119.6, 119.1, 118.8, 113.9, 111.3, 109.7, 31.8. HRMS (ESI) exact mass calcd for C_16_H_14_N_2_ [M+H]^+^ *m*/*z* 233.1073, found 233.1083.

*4-((1-methyl-1H-indol-3-yl)methyl)benzonitrile (**3b**):* The desired pure product was obtained in 73% yield as a white solid. ^1^H NMR (400 MHz, CDCl_3_) δ 7.50 (d, *J* = 8.2 Hz, 2H), 7.39 (d, *J* = 7.9 Hz, 1H), 7.32 (d, *J* = 8.2 Hz, 2H), 7.28 (d, *J* = 8.2 Hz, 1H), 7.23–7.16 (m, 1H), 7.10–7.01 (m, 1H), 6.78 (s, 1H), 4.12 (s, 2H), 3.71 (s, 3H). ^13^C NMR (151 MHz, CDCl_3_) δ 147.3, 137.3, 132.2, 129.4, 127.6, 127.4, 121.9, 119.2, 119.1, 119.0, 112.4, 109.7, 109.4, 32.7, 31.7. HRMS (ESI) exact mass calcd for C_17_H_15_N_2_ [M+H]^+^ *m*/*z* 247.1235, found 247.1240.

*4-((2-methyl-1H-indol-3-yl)methyl)benzonitrile (**3c**):* The desired pure product was obtained in 76% yield as a white solid. ^1^H NMR (400 MHz, CDCl_3_) δ 7.87 (s, 1H), 7.45 (d, *J* = 8.1 Hz, 2H), 7.31–7.21 (m, 4H), 7.10 (t, *J* = 7.5 Hz, 1H), 7.02 (t, *J* = 7.4 Hz, 1H), 4.06 (s, 2H), 2.31 (s, 3H). ^13^C NMR (151 MHz, CDCl_3_) δ 147.5, 135.4, 132.2, 132.2, 129.1, 128.5, 121.3, 119.5, 119.3, 118.0, 110.5, 109.4, 108.9, 30.4, 11.7. HRMS (ESI) exact mass calcd for C_17_H_15_N_2_ [M+H]^+^ *m*/*z* 247.1235, found 247.1229.

*4-((5-methoxy-1H-indol-3-yl)methyl)benzonitrile (**3d**):* The desired pure product was obtained in 75% yield as a white solid. ^1^H NMR (600 MHz, CDCl_3_) δ 7.98 (s, 1H), 7.54 (d, *J* = 8.2 Hz, 2H), 7.36 (d, *J* = 8.2 Hz, 2H), 7.26 (d, *J* = 8.8 Hz, 1H), 6.93 (d, *J* = 0.9 Hz, 1H), 6.89–6.85 (m, 1H), 6.84 (d, *J* = 2.3 Hz, 1H), 4.13 (s, 2H), 3.79 (s, 3H). ^13^C NMR (151 MHz, CDCl_3_) δ 154.1, 146.9, 132.2, 131.6, 129.3, 127.5, 123.4, 119.1, 113.6, 112.3, 112.0, 109.7, 100.9, 55.9, 31.8. HRMS (ESI) exact mass calcd for C_17_H_15_N_2_O [M+H]^+^ *m*/*z* 263.1184, found 263.1185.

*4-(4-methoxybenzyl)benzonitrile (**3e**):* The desired pure product was obtained in 41% yield as a white solid. ^1^H NMR (400 MHz, CDCl_3_) δ 7.56 (d, *J* = 8.0 Hz, 2H), 7.28 (s, 2H), 7.08 (d, *J* = 8.3 Hz, 2H), 6.85 (d, *J* = 8.4 Hz, 2H), 3.97 (s, 2H), 3.79 (s, 3H). ^13^C NMR (151 MHz, CDCl_3_) δ 158.3, 147.2, 132.2, 131.4, 129.9, 129.5, 119.0, 114.1, 109.9, 55.3, 41.1. HRMS (ESI) exact mass calcd for C_15_H_14_NO [M+H]^+^ *m*/*z* 224.1075, found 224.1079.

*4-(3,4-dimethoxybenzyl)benzonitrile (**3f**):* The desired pure product was obtained in 83% yield as a white solid. ^1^H NMR (400 MHz, CDCl_3_) δ 7.57 (d, *J* = 8.1 Hz, 2H), 7.28 (d, *J* = 7.8 Hz, 2H), 6.82 (d, *J* = 8.1 Hz, 1H), 6.72–6.69 (m, 1H), 6.66 (s, 1H), 3.98 (s, 2H), 3.86 (s, 3H), 3.83 (s, 3H). ^13^C NMR (151 MHz, CDCl_3_) δ 149.1, 147.8, 147.0, 132.2, 131.8, 129.5, 121.0, 119.0, 112.2, 111.4, 110.0, 55.9, 55.9, 41.5. HRMS (ESI) exact mass calcd for C_16_H_16_NO_2_ [M+H]^+^ *m*/*z* 254.1181, found 254.1187.

*4-(benzo[d][1,3]dioxol-5-ylmethyl)benzonitrile (**3g**):* The desired pure product was obtained in 79% yield as a white solid. ^1^H NMR (400 MHz, CDCl_3_) δ 7.56 (d, *J* = 7.9 Hz, 2H), 7.26 (d, *J* = 7.8 Hz, 2H), 6.75 (d, *J* = 7.8 Hz, 1H), 6.62 (d, *J* = 11.4 Hz, 2H), 5.92 (s, 2H), 3.93 (s, 2H). ^13^C NMR (151 MHz, CDCl_3_) δ 147.9, 146.9, 146.3, 133.0, 132.3, 129.5, 121.9, 119.0, 110.1, 109.3, 108.4, 101.0, 41.6. HRMS (ESI) exact mass calcd for C_15_H_12_NO_2_ [M+H]^+^ *m*/*z* 238.0868, found 238.0859.

*4-(1-(6-methoxynaphthalen-2-yl)ethyl)benzonitrile (**3h**):* The desired pure product was obtained in 36% yield as a white solid. ^1^H NMR (400 MHz, CDCl_3_) δ 7.70 (d, *J* = 8.9 Hz, 1H), 7.66 (d, *J* = 8.5 Hz, 1H), 7.60 (s, 1H), 7.57 (d, *J* = 8.0 Hz, 2H), 7.35 (d, *J* = 8.1 Hz, 2H), 7.20 (d, *J* = 8.9 Hz, 1H), 7.17–7.13 (m, 1H), 7.11 (s, 1H), 4.33 (q, *J* = 7.2 Hz, 1H), 3.91 (s, 3H), 1.72 (d, *J* = 7.2 Hz, 3H). ^13^C NMR (151 MHz, CDCl_3_) δ 157.6, 152.0, 139.7, 133.3, 132.2, 129.2, 128.9, 128.5, 127.2, 126.8, 125.4, 119.0, 109.9, 105.6, 55.3, 44.8, 21.4. HRMS (ESI) exact mass calcd for C_20_H_18_NO [M+H]^+^ *m*/*z* 288.1388, found 288.1382.

### 3.3. General Procedure for Light-Promoted Decarboxylative Cross-Coupling Reaction between 3-Indoleacetic Acid and Nitriles

3-indoleacetic acid (**1a**, 0.2 mmol), nitriles (**2**, 0.1 mmol), K_2_CO_3_ (1 equiv.) and *fac*-Ir(ppy)_3_ (2 mol%) were successively added to a dried reaction tube (10 mL) with a magnetic stirring bar. Air was then withdrawn and backfilled with argon 3 times. Subsequently, degassed DMSO (1 mL) was injected into the tube by syringe. Then, the resulting reaction mixture was performed at room temperature under blue LED (6 W) irradiation for 3 h. The reaction progress was monitored by TLC. After the reactions were completed, the reaction mixture was diluted with water (10 mL) and washed with EA (3 × 10 mL). The combined organic layers were dried over anhydrous Na_2_SO_4_ and concentrated under reduced pressure. The residue was purified by column chromatography to afford the desired compounds **4** (ethylacetate/n-Hexane = 1:3 to 1:10).

*4-((1H-indol-3-yl)methyl)-2-methylbenzonitrile (**4a**) and 4-((1H-indol-3-yl)methyl)-3-methylbenzonitrile (4a’):* The desired pure product was obtained in 80% yield as a white solid (rr = 4:5). ^1^H NMR (400 MHz, CDCl_3_) δ 8.07 (s, 1H), 7.53–7.39 (m, 2H), 7.36 (d, *J* = 3.6 Hz, 1H), 7.26–7.11 (m, 3H), 7.11–7.02 (m, 1H), 6.94 (s, 0.4H), 6.75 (s, 0.5H), 4.10 (d, *J* = 6.7 Hz, 2H), 2.46 (s, 1.3H), 2.35 (s, 1.7H). ^13^C NMR (151 MHz, CDCl_3_) δ 146.9, 145.1, 141.9, 137.9, 136.5, 133.4, 132.5, 130.5, 129.9, 129.8, 127.2, 127.2, 126.6, 122.6, 122.3, 122.3, 119.6, 119.4, 118.9, 118.8, 118.5, 114.0, 113.2, 111.3, 111.3, 110.1, 109.8, 31.7, 29.5, 20.5, 19.4. HRMS (ESI) exact mass calcd for C_17_H_15_N_2_ [M+H]^+^ *m*/*z* 247.1235, found 247.1240.

*3-(pyridin-4-ylmethyl)-1H-indole (**4b**):* The desired pure product was obtained in 72% yield as a white solid. ^1^H NMR (400 MHz, CDCl_3_) δ 8.85 (s, 1H), 8.47 (d, *J* = 5.0 Hz, 2H), 7.43 (d, *J* = 7.8 Hz, 1H), 7.34 (d, *J* = 8.1 Hz, 1H), 7.18 (t, *J* = 6.7 Hz, 3H), 7.07 (t, *J* = 7.4 Hz, 1H), 6.96 (s, 1H), 4.10 (s, 2H). ^13^C NMR (151 MHz, CDCl_3_) δ 150.8, 149.4, 136.6, 127.2, 124.2, 122.9, 122.2, 119.5, 118.8, 113.0, 111.4, 31.1. HRMS (ESI) exact mass calcd for C_14_H_13_N_2_ [M+H]^+^ *m*/*z* 209.1079, found 209.1082.

*3-((2-fluoropyridin-4-yl)methyl)-1H-indole (**4c**):* The desired pure product was obtained in 71% yield as a white solid. ^1^H NMR (400 MHz, CDCl_3_) δ 1H NMR (400 MHz, CDCl3) δ 8.18 (s, 1H), 8.08 (d, *J* = 5.1 Hz, 1H), 7.46–7.31 (m, 2H), 7.24–7.19 (m, 1H), 7.14–7.06 (m, 2H), 7.02 (s, 1H), 6.80 (s, 1H), 4.14 (s, 2H). ^13^C NMR (151 MHz, CDCl_3_) δ 164.2 (d, *J* = 238.2 Hz), 156.6 (d, *J* = 7.6 Hz), 147.2 (d, *J* = 15.1 Hz), 136.4, 127.0, 122.7, 122.5, 121.7 (d, *J* = 3.9 Hz), 119.8, 118.7, 112.6, 111.3, 109.2 (d, *J* = 37.0 Hz), 30.9, 30.9. HRMS (ESI) exact mass calcd for C_14_H_12_FN_2_ [M+H]^+^ *m*/*z* 227.0985, found 227.0989.

*3-((2-chloropyridin-4-yl)methyl)-1H-indole (**4d**):* The desired pure product was obtained in 74% yield as a white solid. ^1^H NMR (400 MHz, CDCl_3_) δ 8.31 (s, 1H), 8.24 (d, *J* = 5.1 Hz, 1H), 7.47–7.36 (m, 2H), 7.22 (t, *J* = 7.5 Hz, 2H), 7.15–7.06 (m, 2H), 7.00 (s, 1H), 4.09 (s, 2H). ^13^C NMR (151 MHz, CDCl_3_) δ 154.0, 151.6, 149.4, 136.4, 126.9, 124.2, 122.8, 122.8, 122. 5, 119.8, 118.7, 112.5, 111.3, 30.8. HRMS (ESI) exact mass calcd for C_14_H_12_ClN_2_ [M+H]^+^ *m*/*z* 243.0689, found 243.0683.

*3-((3-chloropyridin-4-yl)methyl)-1H-indole (**4e**):* The desired pure product was obtained in 79% yield as a white solid. ^1^H NMR (400 MHz, CDCl_3_) δ 8.59 (s, 1H), 8.55 (d, *J* = 2.3 Hz, 1H), 8.30 (d, *J* = 5.0 Hz, 1H), 7.48 (d, *J* = 7.9 Hz, 1H), 7.39 (d, *J* = 8.1 Hz, 1H), 7.22 (d, *J* = 7.2 Hz, 1H), 7.16–7.10 (m, 1H), 7.08 (d, *J* = 5.0 Hz, 1H), 7.03 (d, *J* = 2.1 Hz, 1H), 4.23 (s, 2H). ^13^C NMR (151 MHz, CDCl_3_) δ 148.9, 148.0, 147.5, 136.4, 132.1, 127.1, 125.0, 123.2, 122.4, 119.7, 118.8, 111.4, 111.4, 28.5. HRMS (ESI) exact mass calcd for C_14_H_12_ClN_2_ [M+H]^+^ *m*/*z* 243.0689, found 243.0692.

### 3.4. Scale-Up Experiment

3-Indoleacetic acid (**1a**, 15 mmol), 1,4-dicyanobenzene (**2a**, 7.5 mmol), K_2_CO_3_ (7.5 mmol) and *fac*-Ir(ppy)_3_ (1 mol%) were successively added to a dried reaction tube (100 mL) with a magnetic stirring bar. Air was then withdrawn and backfilled with argon 3 times. Subsequently, degassed DMSO (25 mL) was injected into the tube by syringe. Then, the resulting reaction mixture was performed at room temperature under blue LED (6 W) irradiation for 15 h. After the reaction was completed, the reaction mixture was diluted with water (50 mL) and washed with EA (3 × 50 mL). The combined organic layers were dried over anhydrous Na_2_SO_4_ and concentrated under reduced pressure. The residue was purified by column chromatography (ethylacetate/n-Hexane = 1:8) to afford the desired compound **3a** (1.2056 g, 69%).

### 3.5. The Light On/Off Experiment

Following the literature procedure [53], to a dried reaction tube (10 mL) with a magnetic stirring bar were added 3-Indoleacetic acid (**1a**, 0.2 mmol), 1,4-dicyanobenzene (**2a**, 0.1 mmol), K_2_CO_3_ (1 equiv.) and *fac*-Ir(ppy)_3_ (2 mol%) successively. Air was then withdrawn and backfilled with argon 3 times. Subsequently, degassed DMSO (1 mL) was injected into the tube by syringe. Then, the resulting reaction mixture was performed at room temperature under blue LED (6 W) irradiation. eight identical reactions were carried out simultaneously and yield was determined by ^1^H NMR of the crude mixture using 1,3,5-trimethylbenzene (13.9 μL, 0.10 mmol, 0.5 equiv.) as internal standard. The experiments with continuous intervals of irradiation and dark periods led to total interruption of the reaction proceed in the absence of light, and reactivity is restored under further light (The details are in the Supplementary Materials). These results indicated that light is an essential component of the reaction.

## 4. Conclusions

In summary, we have described a photoredox-catalyzed decarboxylative cross-coupling reaction of aryl acetic acids and aryl nitriles achieved under an argon atmosphere in high yields. A tentative radical mechanism has been proposed. Further studies of the detailed reaction mechanism and application are underway in our laboratory.

## Figures and Tables

**Table 1:** Screening of reaction conditions ^a^.

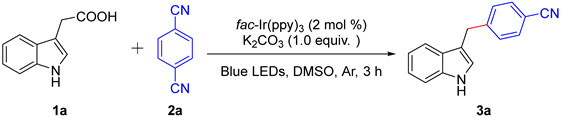		
**Entry**	**Deviation from Standard Conditions**	**3a Yield (%) ^b^**
1	No deviation	82
2	Without LED irradiation	trace
3	Without fac-Ir(ppy)_3_	0
4	[Ir(dtbbpy)(ppy)_2_]PF_6_ instead of fac-Ir(ppy)_3_	32
5	Ru(bpy)_3_(PF_6_)_2_ instead of fac-Ir(ppy)_3_	0
6	EosinY instead of fac-Ir(ppy)_3_	0
7	Rose bengal instead of fac-Ir(ppy)_3_	0
8	without K_2_CO_3_	30
9	K_3_PO_4_ instead of K_2_CO_3_	67
10	NaOAc instead of K_2_CO_3_	64
11	CsF instead of K_2_CO_3_	68
12	DCM instead of DMSO	0
13	MeCN instead of DMSO	61
14	DMA instead of DMSO	67
15	Air instead of Ar	trace

[^a^] Reaction conditions: **1a** (0.2 mmol), **2a** (0.1 mmol), fac-Ir(ppy)_3_ (2 mol%), K_2_CO_3_ (1.0 equiv.), DMSO (1 mL). [^b^] Isolated yields.

**Table 2:** Scope of the decarboxylative cross-coupling reaction of various aryl acetic acids with 1,4-DCB ^a,b^.

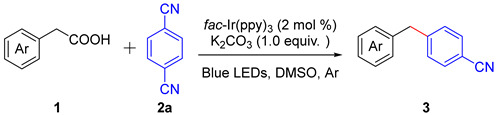
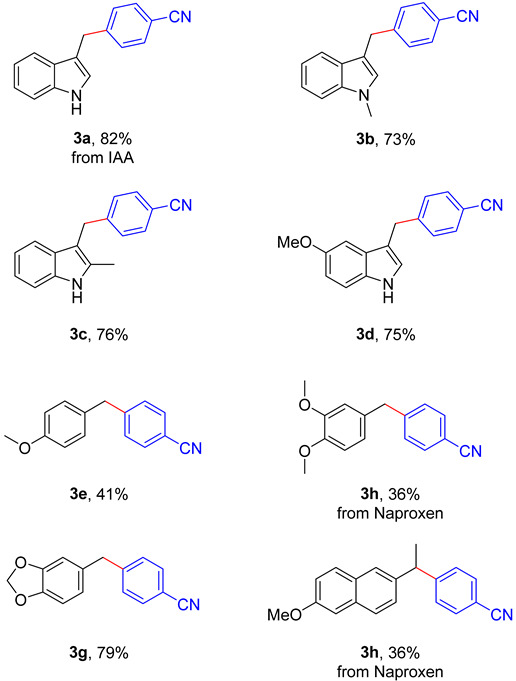

[^a^] Reaction conditions: **1** (0.2 mmol), **2a** (0.1 mmol), fac-Ir(ppy)_3_ (2 mol%), K_2_CO_3_ (1.0 equiv.), DMSO (1 mL), 3–12 h. [^b^] Isolated yields.

**Table 3:** Scope of the decarboxylative cross-coupling reaction of various aryl nitriles with 3-indoleacetic acid ^a,b^.

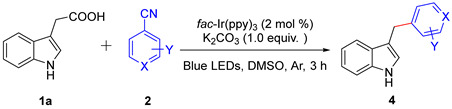
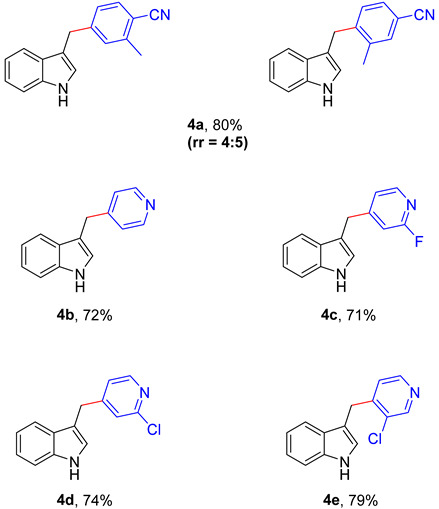

[^a^] Reaction conditions: **1a** (0.2 mmol), **2** (0.1 mmol), fac-Ir(ppy)_3_ (2 mol%), K_2_CO_3_ (1.0 equiv.), DMSO (1 mL). [^b^] Isolated yields.

## Data Availability

The data presented in this study are available in this article.

## Supplementary Materials

The following supporting information can be downloaded at: https://www.mdpi.com/article/10.3390/molecules29092156/s1, Part 1, Experimental Section; Part 2, Scale-up experiment; Part 3, The light on/off experiment; Part 4, Characterizations of compounds; Part 5, NMR spectra of the products.
