# Supplementary material for: Photoredox-Catalyzed Decarboxylative Cross-Coupling Reaction to Synthesis Unsymmetrical Diarylmethanes
Source: Molecules. 2024 May 6;29(9):2156. doi: 10.3390/molecules29092156 (PMC11085496; doi:10.3390/molecules29092156)
Supplement: Supplementary file 1 [file molecules-29-02156-s001.zip › molecules-2984431-supplementary materials.pdf]

Supplementary Materials

Photoredox-Catalyzed Decarboxylative Cross-Coupling Reaction to Synthesis Unsymmetrical Diarylmethanes

Guozhe Guo *, Yuquan Zhang, Yanchun Li and Zhijun Li

Gansu Key Laboratory of Efficient Utilization of Oil and Gas Resources, College of Petroleum and Chemical Engineering, Longdong University, Qingyang 745000, China

* Correspondence: guoguozhe2008@126.com

Table of Contents

Experimental Section……………………………………………………………………(Page S2)

Scale-up experiment……………………………………………………………………(Page S3)

The light on/off experiment…………………………………………………………(Page S3)

Characterizations of compounds…………………………………………………….(Page S5)

NMR spectra of the products……………………………………………………….(Page S10)

Experimental Section

General Information

All materials, reagents and solvents were purchased from commercial suppliers and were used without further purification. Analytical TLC was performed with silica gel GF254 plates, and the products were visualized by UV detection. Flash chromatography was carried out using silica gel 200-300. The ^{1}H NMR (400 or 600 MHz) and ^{13}C NMR (151 MHz) spectra were measured with $CDCl_3$ as solvent. All chemical shifts (δ) are reported in ppm and coupling constants (*J*) in Hz. High resolution mass spectra (HR-MS) were recorded under electrospray ionization (ESI) conditions.

General procedure for Light promoted decarboxylative cross coupling reaction between arylacetic acids and 1,4-dicyanobenzene

To a dried reaction tube (10 mL) with a magnetic stirring bar were added arylacetic acids (**1**, 0.2 mmol), 1,4-dicyanobenzene (**2a**, 0.1 mmol), K_2CO_3 (1 equiv.) and *fac*-Ir(ppy)$_3$ (2 mol %) successively. Air was then withdrawn and backfilled with argon 3 times. Subsequently, degassed DMSO (1 mL) was injected into the tube by syringe. Then, the resulting reaction mixture was performed at room temperature under blue LEDs (6 W) irradiation for 3-24 hours. The reaction progress was monitored by TLC. After the reactions were completed, the reaction mixture was diluted with water (10 mL) and washed with EA (3×10 mL). The combined organic layers dried over anhydrous Na_2SO_4 and concentrated under reduced pressure. The residue was purified by column chromatography to afford the desired compounds **3** (ethylacetate / n-Hexane = 1:8 to 1:100).

General procedure for Light promoted decarboxylative cross coupling reaction between 3-indoleacetic acid and nitriles

To a dried reaction tube (10 mL) with a magnetic stirring bar were added 3-indoleacetic acid (**1a**, 0.2 mmol), nitriles (**2**, 0.1 mmol), K_2CO_3 (1 equiv.) and *fac*-Ir(ppy)$_3$ (2 mol %) successively. Air was then withdrawn and backfilled with argon 3 times. Subsequently, degassed DMSO (1 mL) was injected into the tube by syringe. Then, the resulting reaction mixture was performed at room temperature under blue LEDs (6 W) irradiation for 3 hours. The reaction progress was monitored by TLC. After the reactions were completed, the reaction mixture was diluted with water (10 mL) and washed with EA (3×10 mL). The combined organic layers dried over anhydrous Na_2SO_4 and concentrated under reduced pressure. The residue was purified by column chromatography to afford the desired compounds **4** (ethylacetate / n-Hexane = 1:3 to 1:10).

Scale-up experiment

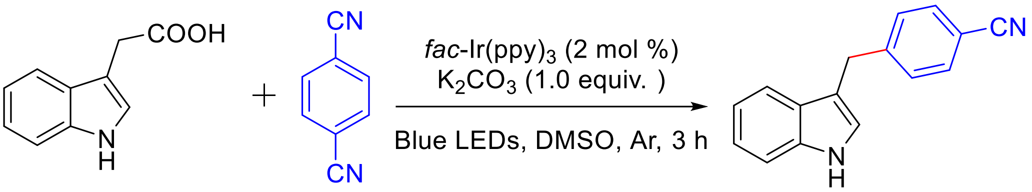

1a, 15 mmol **2a** **3a,** 69% (1.2056 g)

To a dried reaction tube (100 mL) with a magnetic stirring bar were added 3-Indoleacetic acid (**1a**, 15 mmol), terephthalonitrile (**2a**, 7.5 mmol), K_2CO_3 (7.5 mmol) and *fac*-Ir(ppy)$_3$ (1 mol %) successively. Air was then withdrawn and backfilled with argon 3 times. Subsequently, degassed DMSO (25 mL) was injected into the tube by syringe. Then, the resulting reaction mixture was performed at room temperature under blue LEDs (6 W) irradiation for 3 hours. After the reaction was completed, the reaction mixture was diluted with water (50 mL) and washed with EA (3×50 mL). The combined organic layers dried over anhydrous Na_2SO_4 and concentrated under reduced pressure. The residue was purified by column chromatography (ethylacetate / n-Hexane = 1:8) to afford the desired compound **3a** (1.2056 g, 69%).

The light on/off experiment

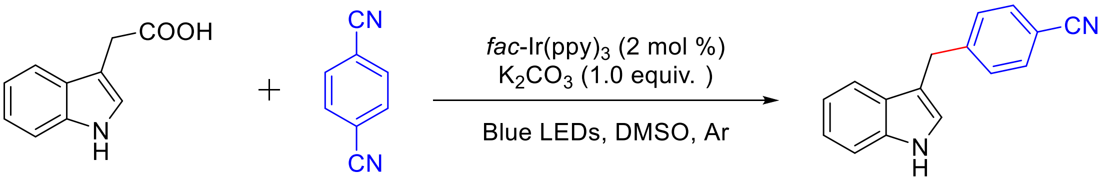

1a **2a** **3a**

Following the literature procedure [1], to a dried reaction tube (10 mL) with a magnetic stirring bar were added carboxylic acids (**1a**, 0.2 mmol), nitriles (**2a**, 0.1 mmol), K_2CO_3 (1 equiv.) and *fac*-Ir(ppy)$_3$ (2 mol %) successively. Air was then withdrawn and backfilled with argon 3 times. Subsequently, degassed DMSO (1 mL) was injected into the tube by syringe. Then, the resulting reaction mixture was performed at room temperature under blue LEDs (6 W) irradiation. eight identical reactions were carried out simultaneously and yield was determined by ^{1}H NMR of the crude mixture using 1,3,5-trimethylbenzene (13.9 μL, 0.10 mmol, 0.5 equiv.) as internal standard. The experiments with continuous intervals of irradiation and dark periods led to total interruption of the reaction proceed in the absence of light, and reactivity is restored under further light. These results indicated that light is an essential component of the reaction.

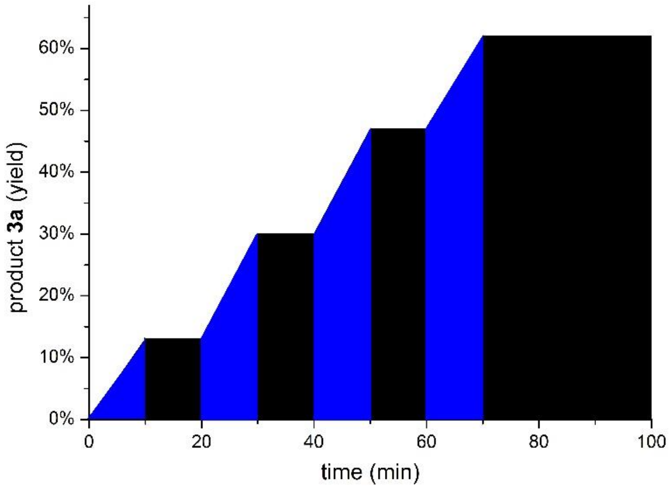

Figure S1: The light on/off experiment

Characterizations of compounds

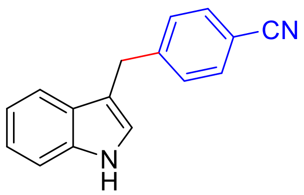

4-((1H-indol-3-yl)methyl)benzonitrile (3a) The desired pure product was obtained in 82% yield as a white solid. ^{1}H NMR (400 MHz, $CDCl_3$) δ 8.08 (s, 1H), 7.60 - 7.52 (m, 2H), 7.47 - 7.32 (m, 4H), 7.25 - 7.16 (m, 1H), 7.14 - 7.04 (m, 1H), 6.98 (d, *J* = 2.3 Hz, 1H), 4.18 (s, 2H). ^{13}C NMR (151 MHz, $CDCl_3$) δ 147.0, 136. 5, 132.2, 129.3, 127.1, 122.6, 122.3, 119.6, 119.1, 118.8, 113.9, 111.3, 109.7, 31.8. HRMS (ESI) exact mass calcd for $C_{16}H_{14}N_2$ $[M+H]^+$ m/z 233.1073, found 233.1083.

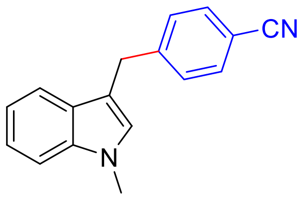

4-((1-methyl-1H-indol-3-yl)methyl)benzonitrile (3b) The desired pure product was obtained in 73% yield as a white solid. ^{1}H NMR (400 MHz, $CDCl_3$) δ 7.50 (d, *J* = 8.2 Hz, 2H), 7.39 (d, *J* = 7.9 Hz, 1H), 7.32 (d, *J* = 8.2 Hz, 2H), 7.28 (d, *J* = 8.2 Hz, 1H), 7.23 - 7.16 (m, 1H), 7.10 - 7.01 (m, 1H), 6.78 (s, 1H), 4.12 (s, 2H), 3.71 (s, 3H). ^{13}C NMR (151 MHz, $CDCl_3$) δ 147.3, 137.3, 132.2, 129.4, 127.6, 127.4, 121.9, 119.2, 119.1, 119.0, 112.4, 109.7, 109.4, 32.7, 31.7. HRMS (ESI) exact mass calcd for $C_{17}H_{15}N_2$ $[M+H]^+$ m/z 247.1235, found 247.1240.

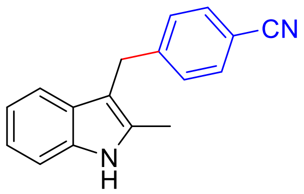

4-((2-methyl-1H-indol-3-yl)methyl)benzonitrile (3c) The desired pure product was obtained in 76% yield as a white solid. ^{1}H NMR (400 MHz, $CDCl_3$) δ 7.87 (s, 1H), 7.45 (d, *J* = 8.1 Hz, 2H), 7.31 - 7.21 (m, 4H), 7.10 (t, *J* = 7.5 Hz, 1H), 7.02 (t, *J* = 7.4 Hz, 1H), 4.06 (s, 2H), 2.31 (s, 3H). ^{13}C NMR (151 MHz, $CDCl_3$) δ 147.5, 135.4, 132.2, 132.2, 129.1, 128.5, 121.3,

119.5, 119.3, 118.0, 110.5, 109.4, 108.9, 30.4, 11.7. HRMS (ESI) exact mass calcd for $C_{17}H_{15}N_2$ $[M+H]^+$ m/z 247.1235, found 247.1229.

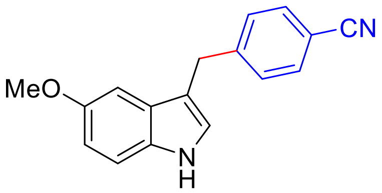

4-((5-methoxy-1H-indol-3-yl)methyl)benzonitrile (3d) The desired pure product was obtained in 75% yield as a white solid. ^{1}H NMR (600 MHz, $CDCl_3$) δ 7.98 (s, 1H), 7.54 (d, *J* = 8.2 Hz, 2H), 7.36 (d, *J* = 8.2 Hz, 2H), 7.26 (d, *J* = 8.8 Hz, 1H), 6.93 (d, *J* = 0.9 Hz, 1H), 6.89 - 6.85 (m, 1H), 6.84 (d, *J* = 2.3 Hz, 1H), 4.13 (s, 2H), 3.79 (s, 3H). ^{13}C NMR (151 MHz, $CDCl_3$) δ 154.1, 146.9, 132.2, 131.6, 129.3, 127.5, 123.4, 119.1, 113.6, 112.3, 112.0, 109.7, 100.9, 55.9, 31.8. HRMS (ESI) exact mass calcd for $C_{17}H_{15}N_2O$ $[M+H]^+$ m/z 263.1184, found 263.1185.

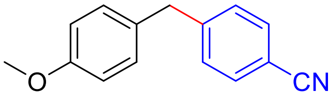

4-(4-methoxybenzyl)benzonitrile (3e) The desired pure product was obtained in 41% yield as a white solid. ^{1}H NMR (400 MHz, $CDCl_3$) δ 7.56 (d, *J* = 8.0 Hz, 2H), 7.28 (s, 2H), 7.08 (d, *J* = 8.3 Hz, 2H), 6.85 (d, *J* = 8.4 Hz, 2H), 3.97 (s, 2H), 3.79 (s, 3H). ^{13}C NMR (151 MHz, $CDCl_3$) δ 158.3, 147.2, 132.2, 131.4, 129.9, 129.5, 119.0, 114.1, 109.9, 55.3, 41.1. HRMS (ESI) exact mass calcd for $C_{15}H_{14}NO$ $[M+H]^+$ m/z 224.1075, found 224.1079.

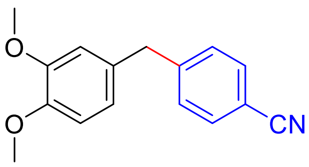

4-(3,4-dimethoxybenzyl)benzonitrile (3f) The desired pure product was obtained in 83% yield as a white solid. ^{1}H NMR (400 MHz, $CDCl_3$) δ 7.57 (d, *J* = 8.1 Hz, 2H), 7.28 (d, *J* = 7.8 Hz, 2H), 6.82 (d, *J* = 8.1 Hz, 1H), 6.72 - 6.69 (m, 1H), 6.66 (s, 1H), 3.98 (s, 2H), 3.86 (s, 3H), 3.83 (s, 3H). ^{13}C NMR (151 MHz, $CDCl_3$) δ 149.1, 147.8, 147.0, 132.2, 131.8, 129.5, 121.0, 119.0, 112.2, 111.4, 110.0, 55.9, 55.9, 41.5. HRMS (ESI) exact mass calcd for $C_{16}H_{16}NO_2$ $[M+H]^+$ m/z 254.1181, found 254.1187.

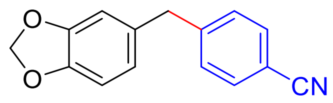

4-(benzo[d][1,3]dioxol-5-ylmethyl)benzonitrile (3g) The desired pure product was obtained in 79% yield as a white solid. ^{1}H NMR (400 MHz, $CDCl_3$) δ 7.56 (d, *J* = 7.9 Hz, 2H), 7.26 (d, *J* = 7.8 Hz, 2H), 6.75 (d, *J* = 7.8 Hz, 1H), 6.62 (d, *J* = 11.4 Hz, 2H), 5.92 (s, 2H), 3.93 (s, 2H). ^{13}C NMR (151 MHz, $CDCl_3$) δ 147.9, 146.9, 146.3, 133.0, 132.3, 129.5, 121.9, 119.0, 110.1, 109.3, 108.4, 101.0, 41.6. HRMS (ESI) exact mass calcd for $C_{15}H_{12}NO_2$ $[M+H]^+$ m/z 238.0868, found 238.0859.

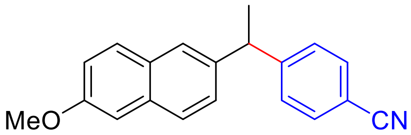

4-(1-(6-methoxynaphthalen-2-yl)ethyl)benzonitrile (3h) The desired pure product was obtained in 36% yield as a white solid. ^{1}H NMR (400 MHz, $CDCl_3$) δ 7.70 (d, *J* = 8.9 Hz, 1H), 7.66 (d, *J* = 8.5 Hz, 1H), 7.60 (s, 1H), 7.57 (d, *J* = 8.0 Hz, 2H), 7.35 (d, *J* = 8.1 Hz, 2H), 7.20 (d, *J* = 8.9 Hz, 1H), 7.17 - 7.13 (m, 1H), 7.11 (s, 1H), 4.33 (q, *J* = 7.2 Hz, 1H), 3.91 (s, 3H), 1.72 (d, *J* = 7.2 Hz, 3H). ^{13}C NMR (151 MHz, $CDCl_3$) δ 157.6, 152.0, 139.7, 133.3, 132.2, 129.2, 128.9, 128.5, 127.2, 126.8, 125.4, 119.0, 109.9, 105.6, 55.3, 44.8, 21.4. HRMS (ESI) exact mass calcd for $C_{20}H_{18}NO$ $[M+H]^+$ m/z 288.1388, found 288.1382.

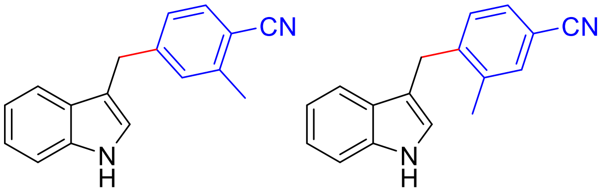

4-((1H-indol-3-yl)methyl)-2-methylbenzonitrile (4a) and 4-((1H-indol-3-yl)methyl)-3-methylbenzonitrile (4a') The desired pure product was obtained in 80% yield as a white solid (rr = 4:5). ^{1}H NMR (400 MHz, $CDCl_3$) δ 8.07 (s, 1H), 7.53 - 7.39 (m, 2H), 7.36 (d, *J* = 3.6 Hz, 1H), 7.26 - 7.11 (m, 3H), 7.11 - 7.02 (m, 1H), 6.94 (s, 0.4H), 6.75 (s, 0.5H), 4.10 (d, *J* = 6.7 Hz, 2H), 2.46 (s, 1.3H), 2.35 (s, 1.7H). ^{13}C NMR (151 MHz, $CDCl_3$) δ 146.9, 145.1, 141.9, 137.9, 136.5, 133.4, 132.5, 130.5, 129.9, 129.8, 127.2, 127.2, 126.6, 122.6, 122.3, 122.3, 119.6, 119.4, 118.9, 118.8, 118.5, 114.0, 113.2, 111.3, 111.3, 110.1, 109.8, 31.7, 29.5, 20.5, 19.4. HRMS (ESI) exact mass calcd for $C_{17}H_{15}N_2$ $[M+H]^+$ m/z 247.1235, found 247.1240.

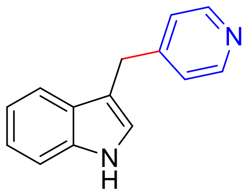

3-(pyridin-4-ylmethyl)-1H-indole (4b) The desired pure product was obtained in 72% yield as a white solid. ^{1}H NMR (400 MHz, $CDCl_3$) δ 8.85 (s, 1H), 8.47 (d, *J* = 5.0 Hz, 2H), 7.43 (d, *J* = 7.8 Hz, 1H), 7.34 (d, *J* = 8.1 Hz, 1H), 7.18 (t, *J* = 6.7 Hz, 3H), 7.07 (t, *J* = 7.4 Hz, 1H), 6.96 (s, 1H), 4.10 (s, 2H). ^{13}C NMR (151 MHz, $CDCl_3$) δ 150.8, 149.4, 136.6, 127.2, 124.2, 122.9, 122.2, 119.5, 118.8, 113.0, 111.4, 31.1. HRMS (ESI) exact mass calcd for $C_{14}H_{13}N_2$ $[M+H]^+$ m/z 209.1079, found 209.1082.

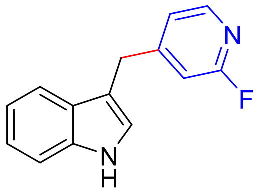

3-((2-fluoropyridin-4-yl)methyl)-1H-indole (4c) The desired pure product was obtained in 71% yield as a white solid. ^{1}H NMR (400 MHz, $CDCl_3$) δ 8.18 (s, 1H), 8.08 (d, *J* = 5.1 Hz, 1H), 7.46 - 7.31 (m, 2H), 7.24 - 7.19 (m, 1H), 7.14 - 7.06 (m, 2H), 7.02 (s, 1H), 6.80 (s, 1H), 4.14 (s, 2H). ^{13}C NMR (151 MHz, $CDCl_3$) δ 164.2 (d, *J* = 238.2 Hz), 156.6 (d, *J* = 7.6 Hz), 147.2 (d, *J* = 15.1 Hz), 136.4, 127.0, 122.7, 122.5, 121.7 (d, *J* = 3.9 Hz), 119.8, 118.7, 112.6, 111.3, 109.2 (d, *J* = 37.0 Hz), 30.9, 30.9. HRMS (ESI) exact mass calcd for $C_{14}H_{12}FN_2$ $[M+H]^+$ m/z 227.0985, found 227.0989.

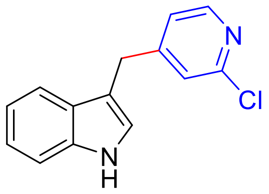

3-((2-chloropyridin-4-yl)methyl)-1H-indole (4d) The desired pure product was obtained in 74% yield as a white solid. ^{1}H NMR (400 MHz, $CDCl_3$) δ 8.31 (s, 1H), 8.24 (d, *J* = 5.1 Hz, 1H), 7.47 - 7.36 (m, 2H), 7.22 (t, *J* = 7.5 Hz, 2H), 7.15 - 7.06 (m, 2H), 7.00 (s, 1H), 4.09 (s, 2H). ^{13}C NMR (151 MHz, $CDCl_3$) δ 154.0, 151.6, 149.4, 136.4, 126.9, 124.2, 122.8, 122.8, 122. 5, 119.8, 118.7, 112.5, 111.3, 30.8. HRMS (ESI) exact mass calcd for $C_{14}H_{12}ClN_2$ $[M+H]^+$ m/z 243.0689, found 243.0683.

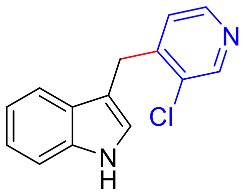

3-((3-chloropyridin-4-yl)methyl)-1H-indole (4e) The desired pure product was obtained in 79% yield as a white solid. ^{1}H NMR (400 MHz, $CDCl_3$) δ 8.59 (s, 1H), 8.55 (d, *J* = 2.3 Hz, 1H), 8.30 (d, *J* = 5.0 Hz, 1H), 7.48 (d, *J* = 7.9 Hz, 1H), 7.39 (d, *J* = 8.1 Hz, 1H), 7.22 (d, *J* = 7.2 Hz, 1H), 7.16 - 7.10 (m, 1H), 7.08 (d, *J* = 5.0 Hz, 1H), 7.03 (d, *J* = 2.1 Hz, 1H), 4.23 (s, 2H). ^{13}C NMR (151 MHz, $CDCl_3$) δ 148.9, 148.0, 147.5, 136.4, 132.1, 127.1, 125.0, 123.2, 122.4, 119.7, 118.8, 111.4, 111.4, 28.5. HRMS (ESI) exact mass calcd for $C_{14}H_{12}ClN_2$ $[M+H]^+$ m/z 243.0689, found 243.0692.

NMR spectra of the products

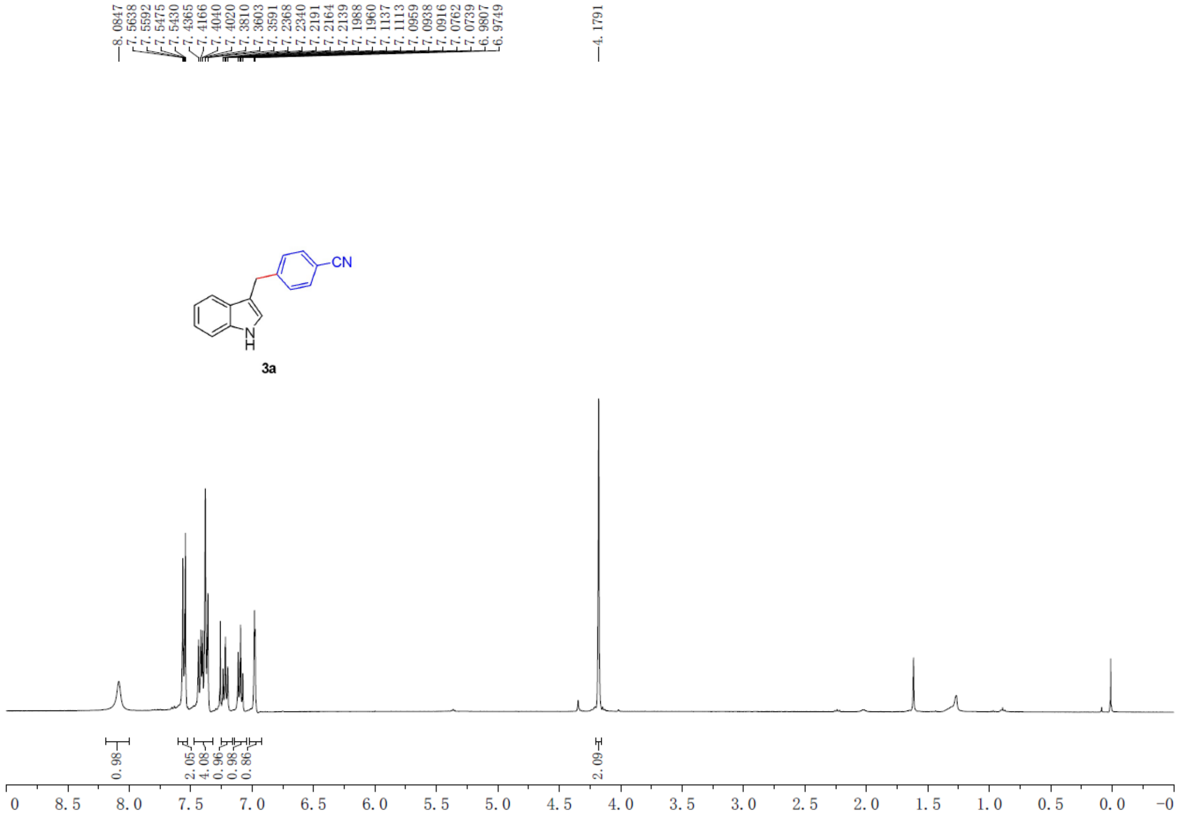

Figure S2: The ^{1}H NMR (400 MHz, $CDCl_3$) of compound **3a**

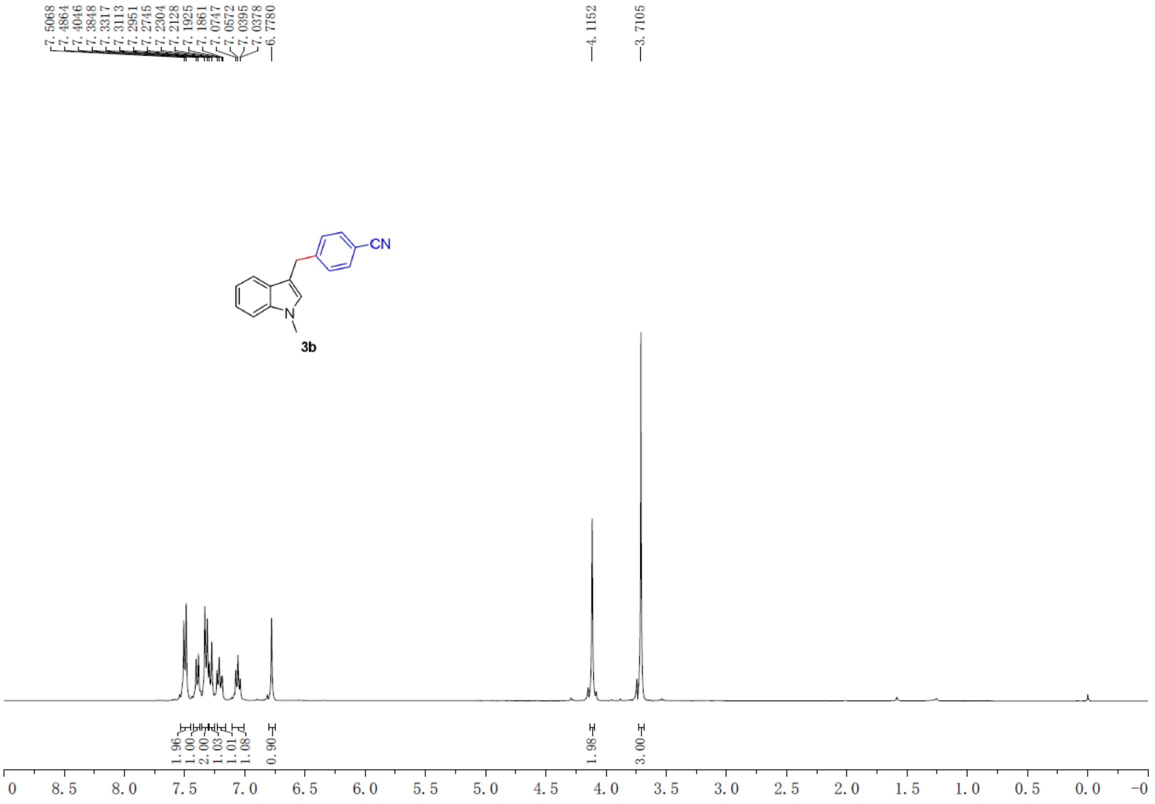

Figure S3: The ^{1}H NMR (400 MHz, $CDCl_3$) of compound **3b**

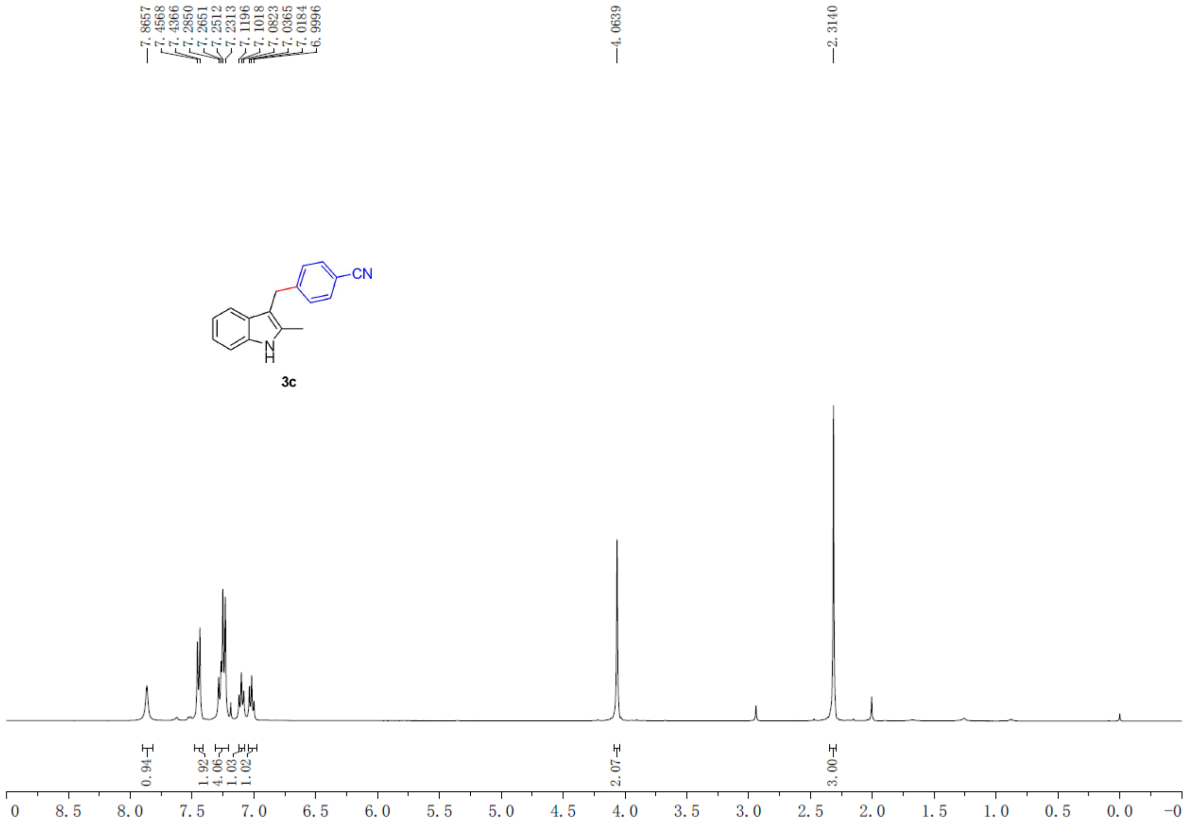

Figure S4: The ^{1}H NMR (400 MHz, $CDCl_3$) of compound **3c**

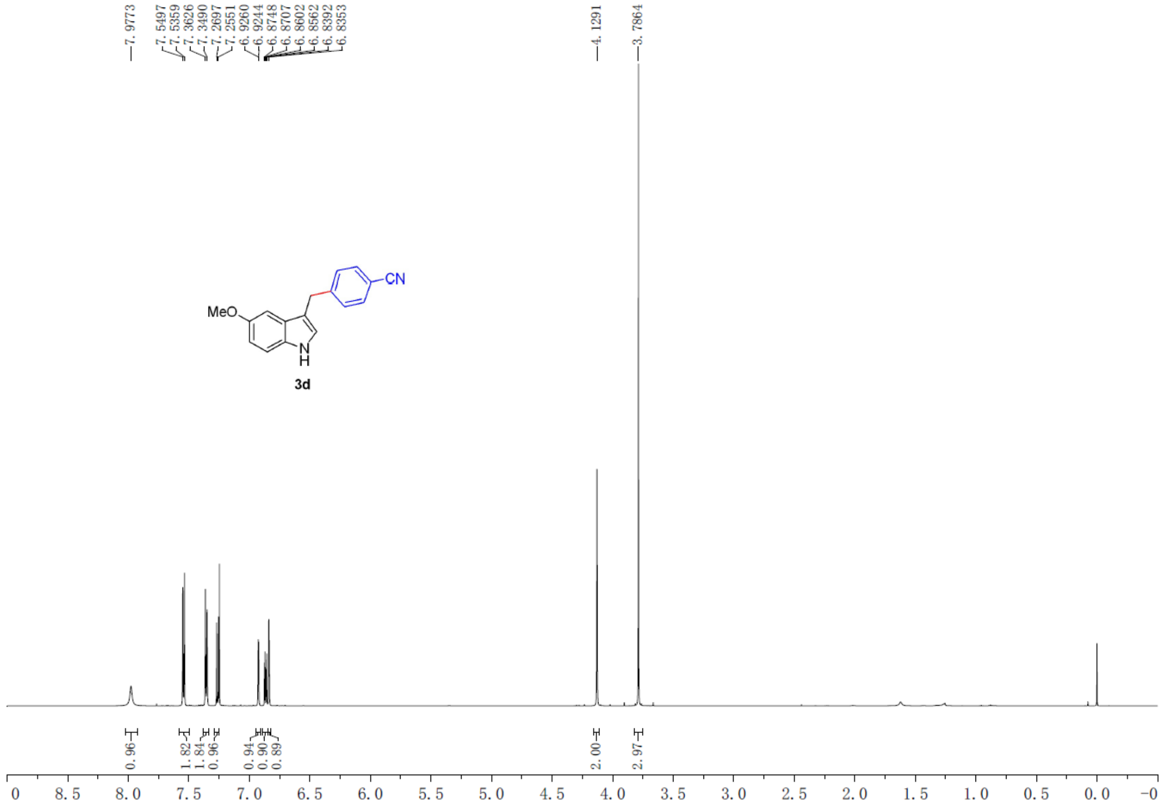

Figure S5: The ^{1}H NMR (600 MHz, $CDCl_3$) of compound **3d**

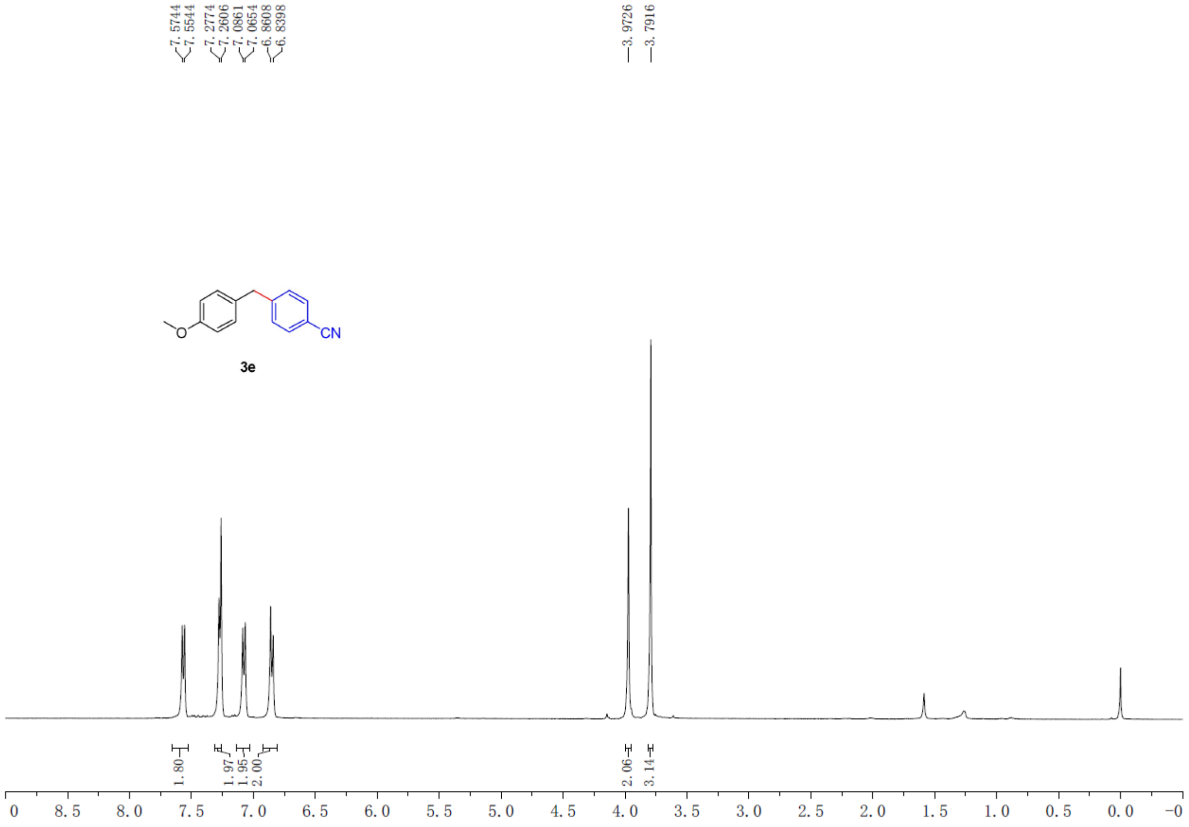

Figure S6: The ¹H NMR (400 MHz, $CDCl_3$) of compound **3e**

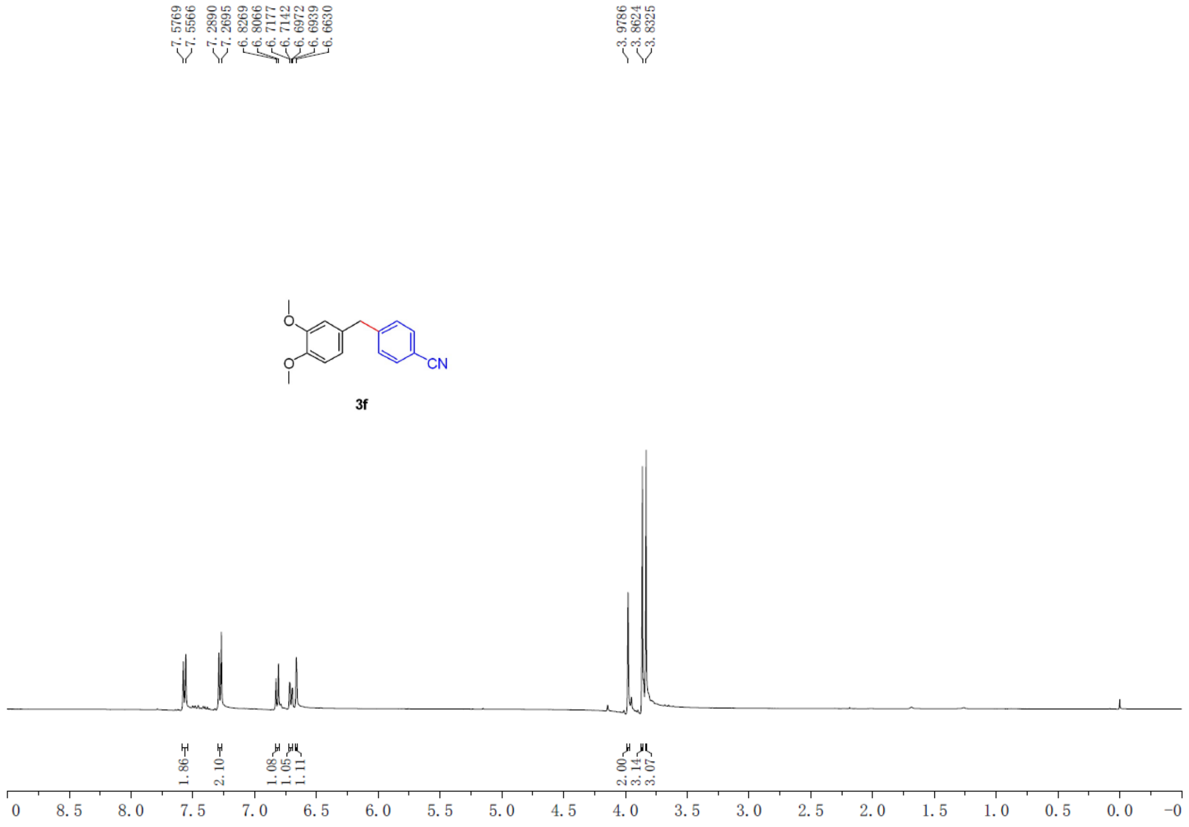

Figure S7: The ¹H NMR (400 MHz, $CDCl_3$) of compound **3f**

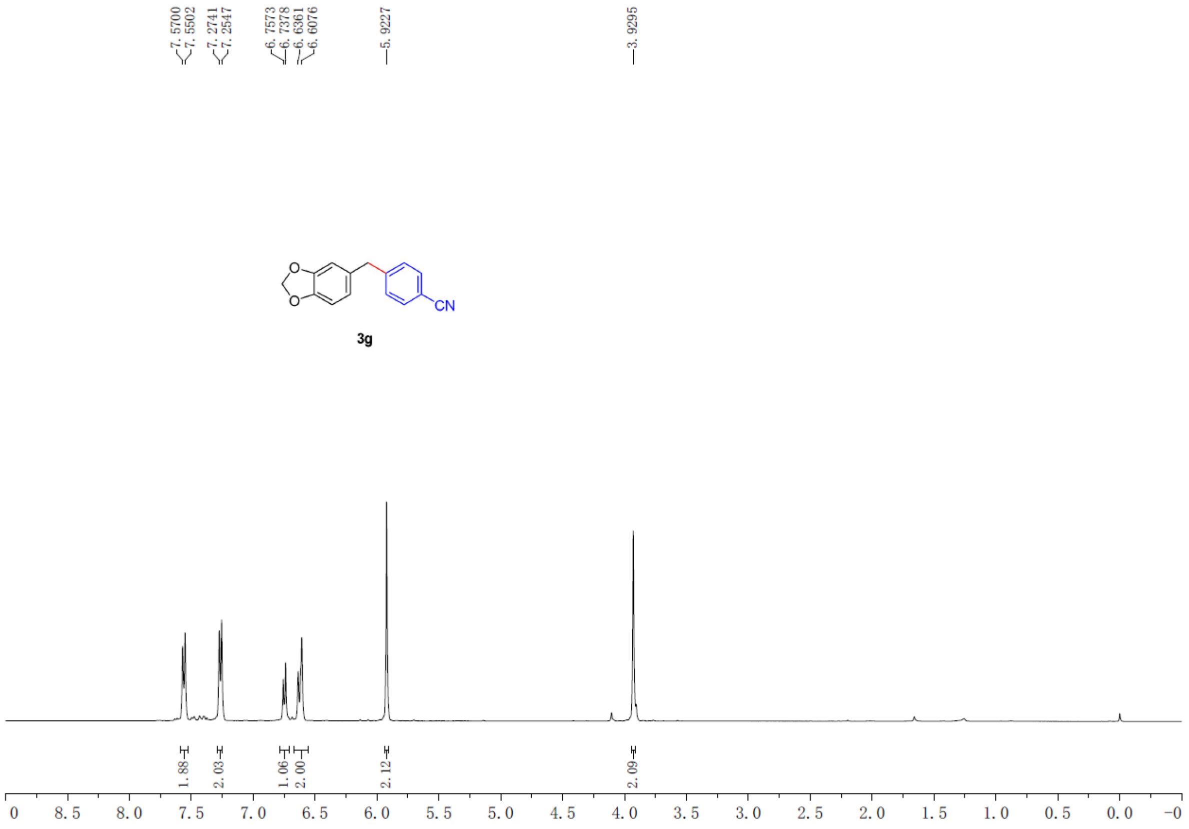

Figure S8: The ^{1}H NMR (400 MHz, $CDCl_3$) of compound **3g**

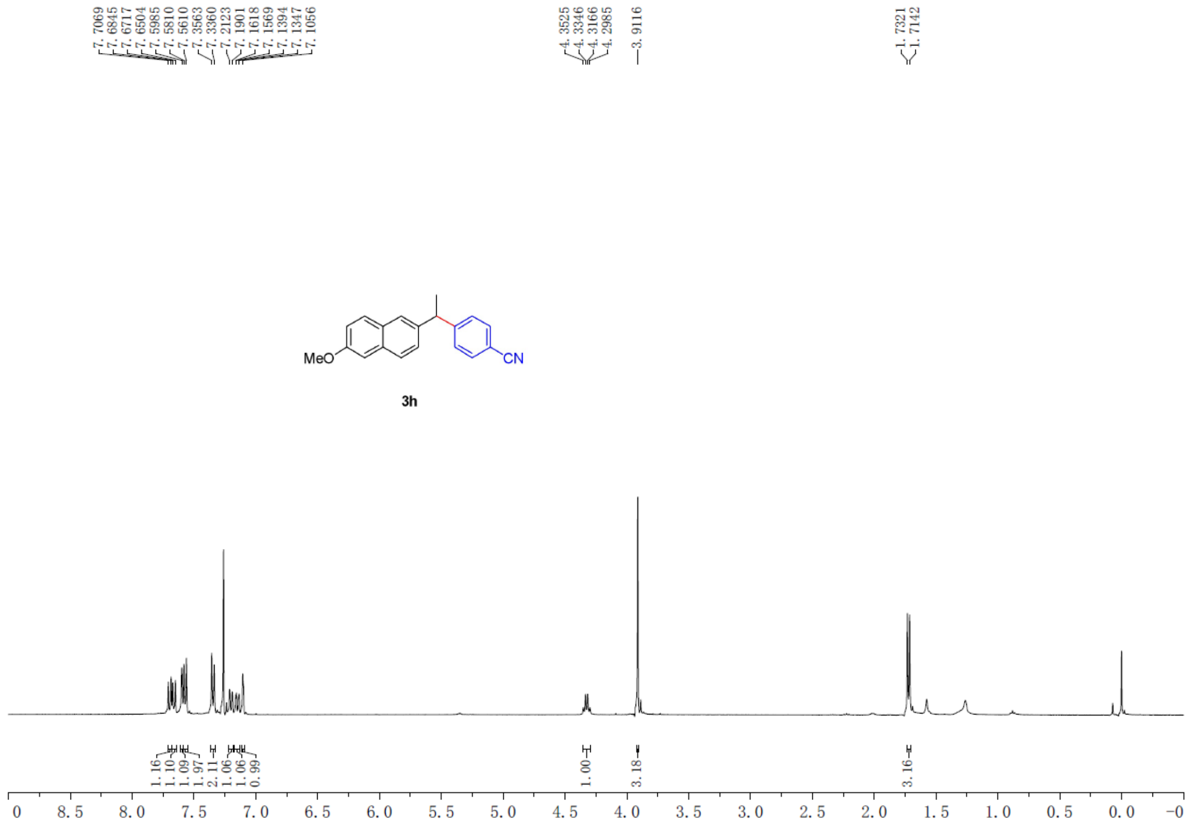

Figure S9: The ^{1}H NMR (400 MHz, $CDCl_3$) of compound **3h**

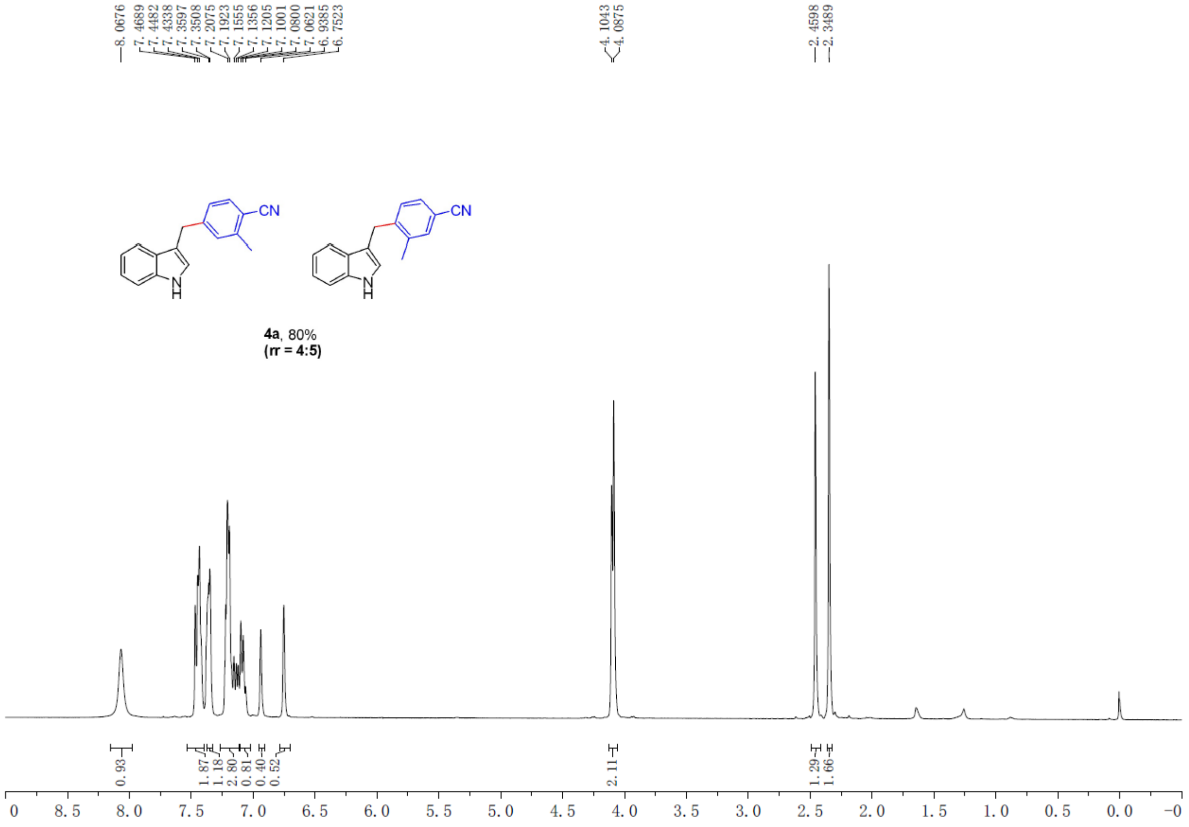

Figure S10: The ^{1}H NMR (400 MHz, $CDCl_3$) of compound **4a**

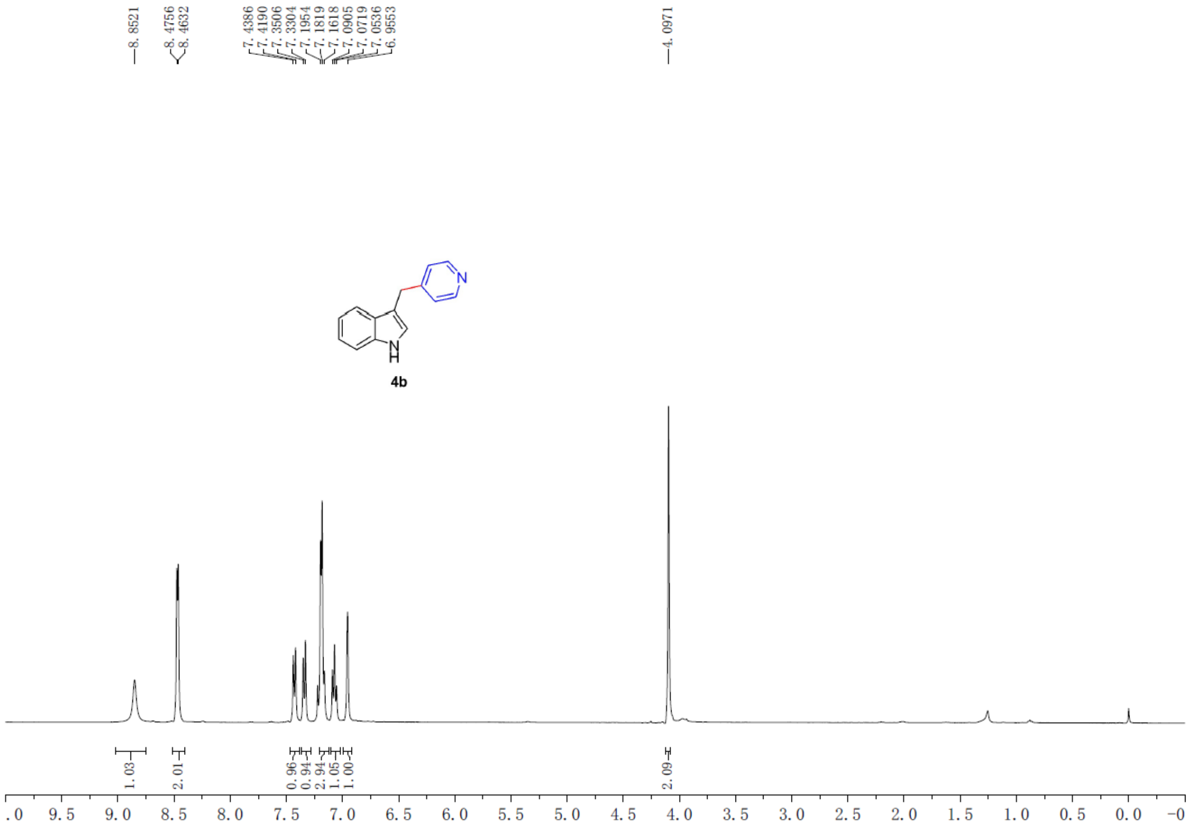

Figure S11: The ^{1}H NMR (400 MHz, $CDCl_3$) of compound **4b**

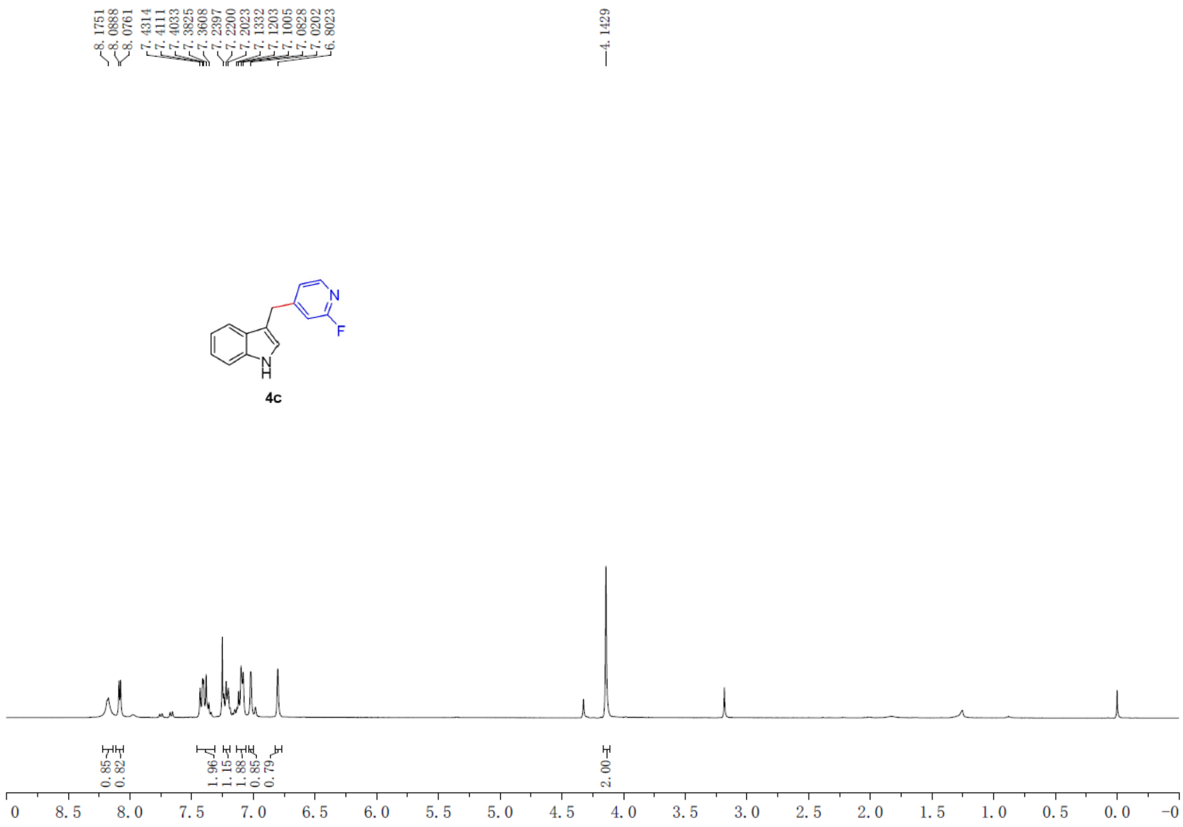

Figure S12: The ^{1}H NMR (400 MHz, $CDCl_3$) of compound **4c**

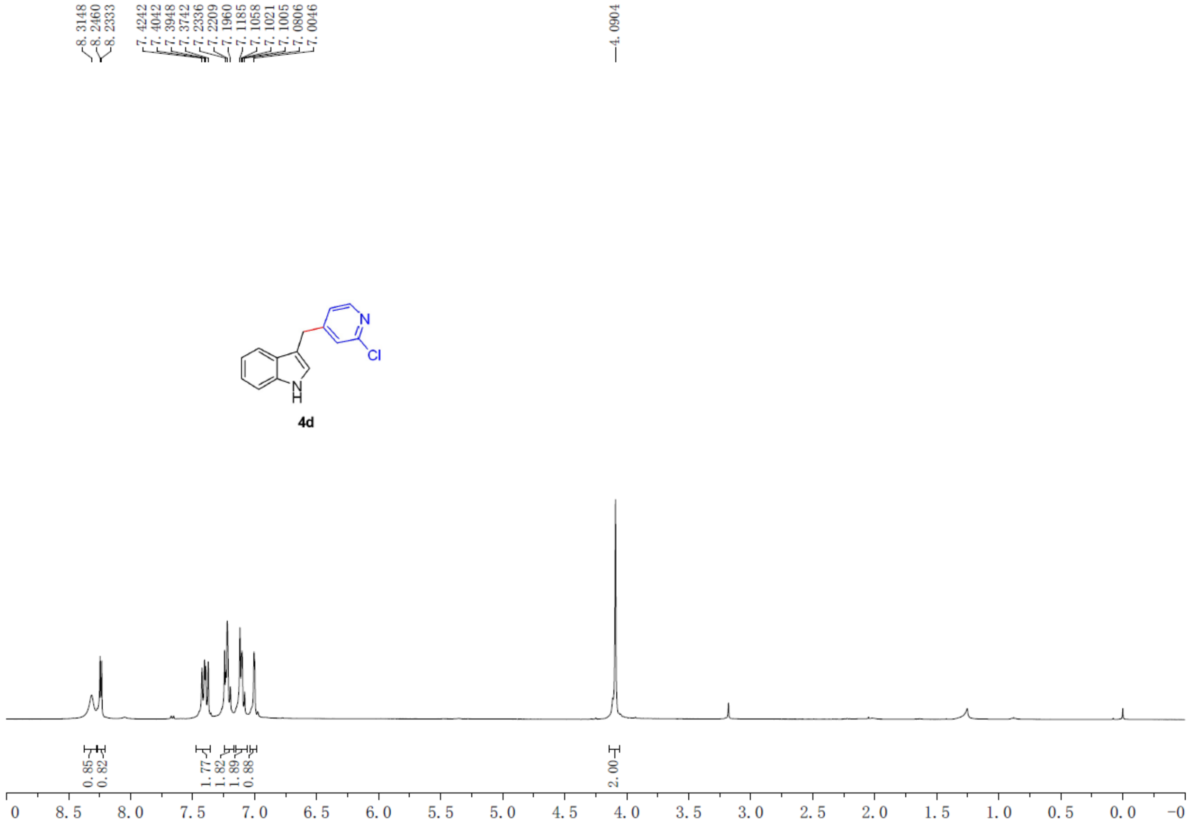

Figure S13: The ^{1}H NMR (400 MHz, $CDCl_3$) of compound **4d**

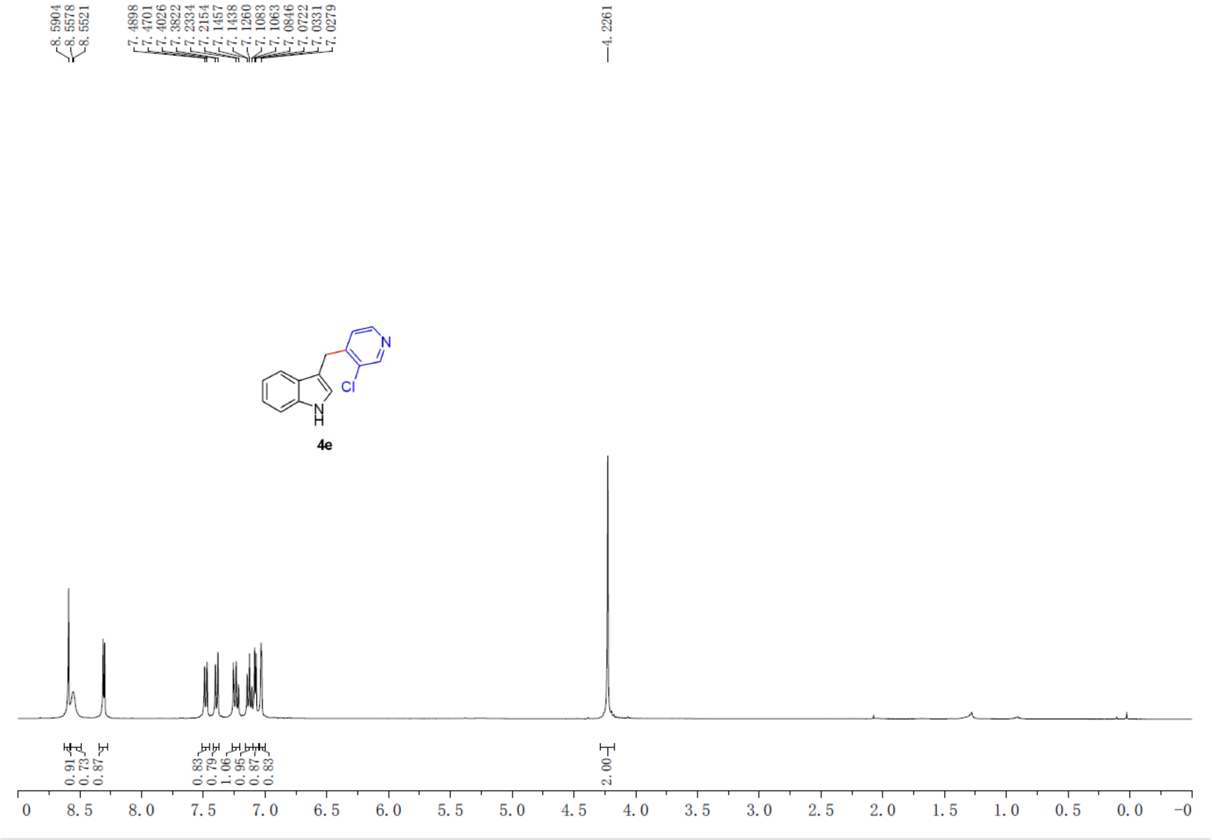

Figure S14: The ^{1}H NMR (400 MHz, $CDCl_3$) of compound **4e**

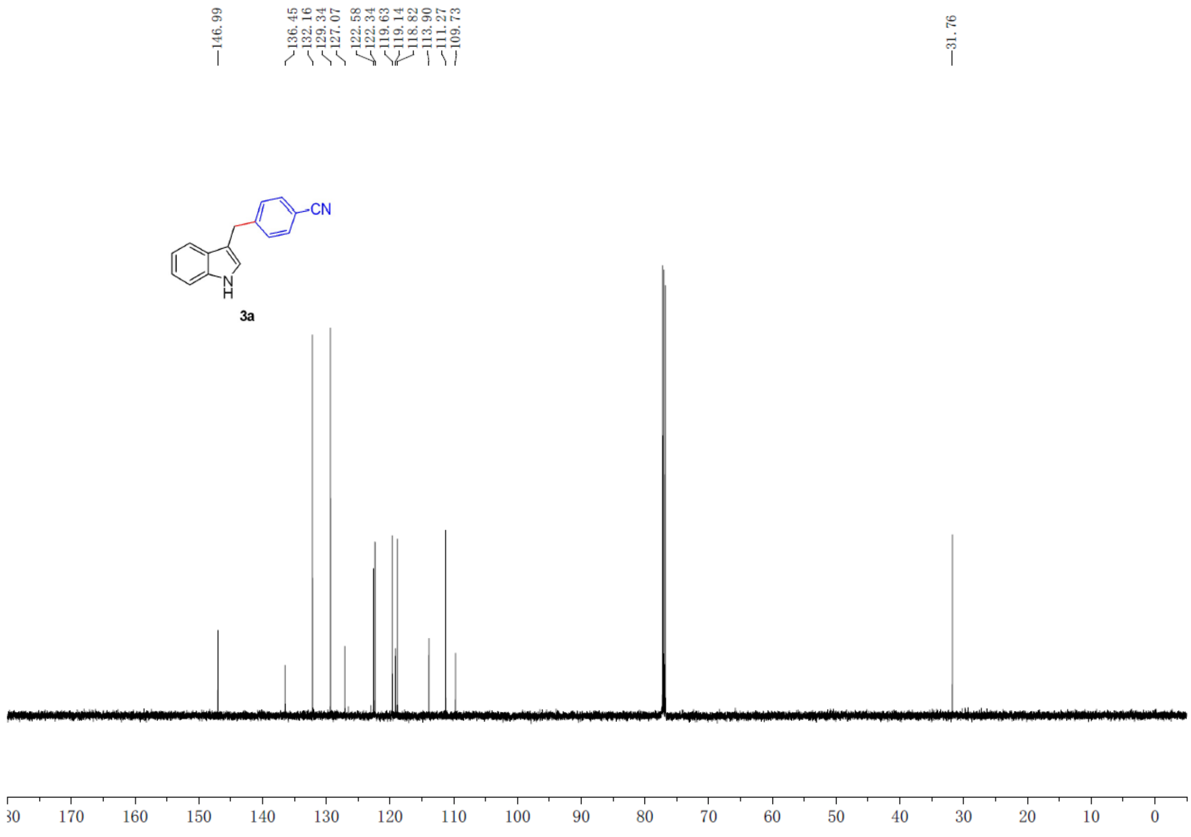

Figure S15: The ^{13}C NMR (151 MHz, $CDCl_3$) of compound **3a**

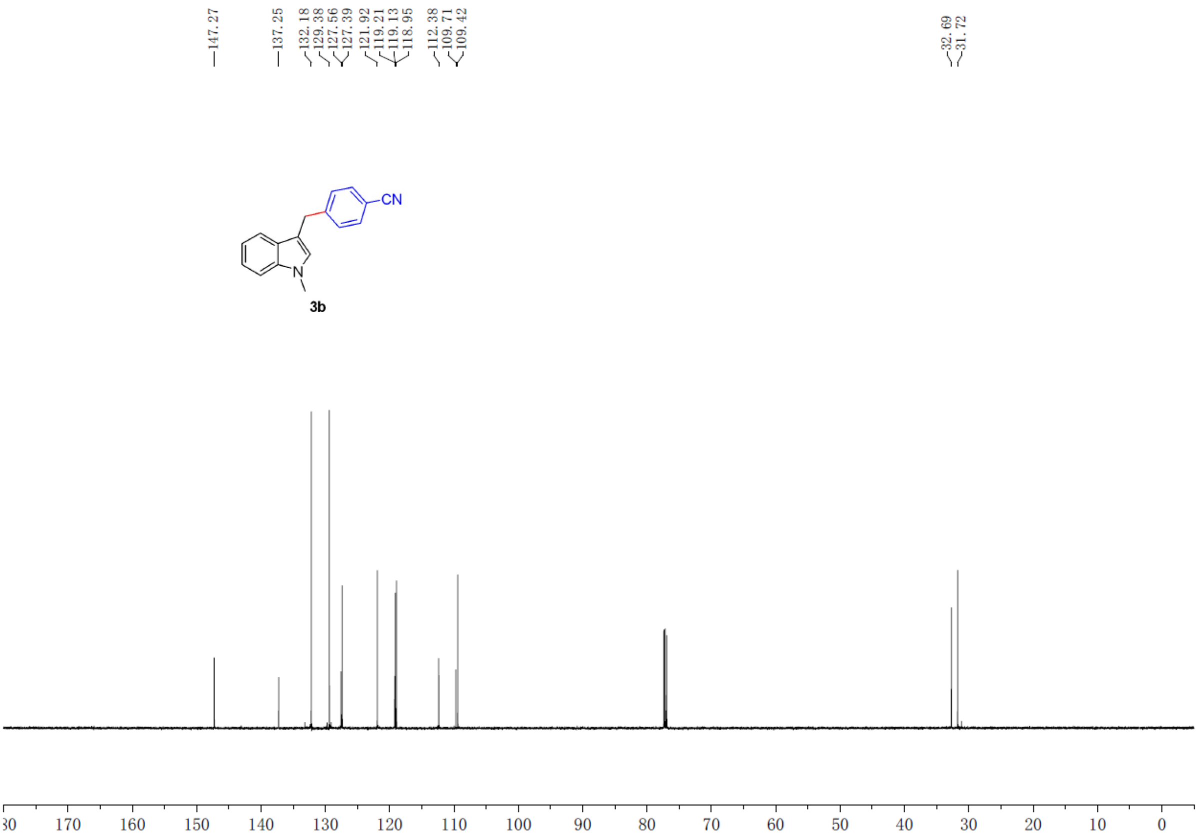

Figure S16: The ^{13}C NMR (151 MHz, $CDCl_3$) of compound **3b**

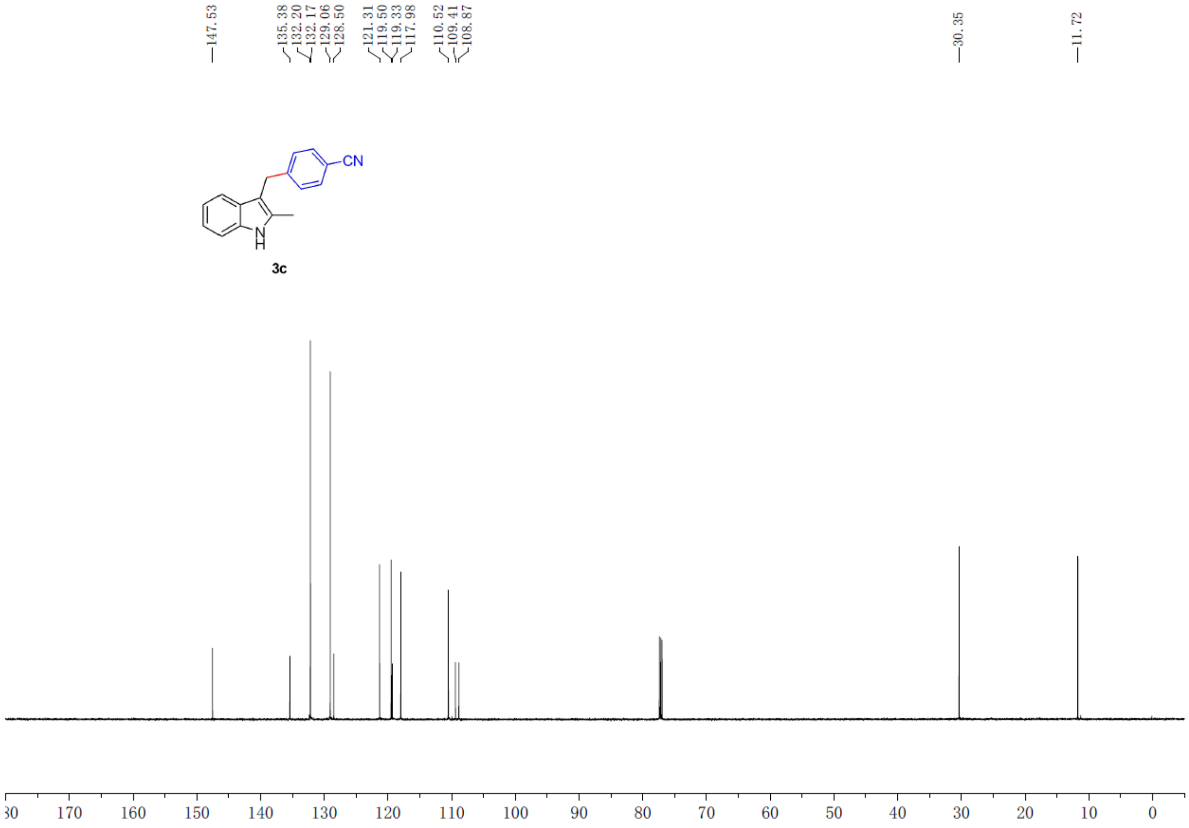

Figure S17: The ^{13}C NMR (151 MHz, $CDCl_3$) of compound **3c**

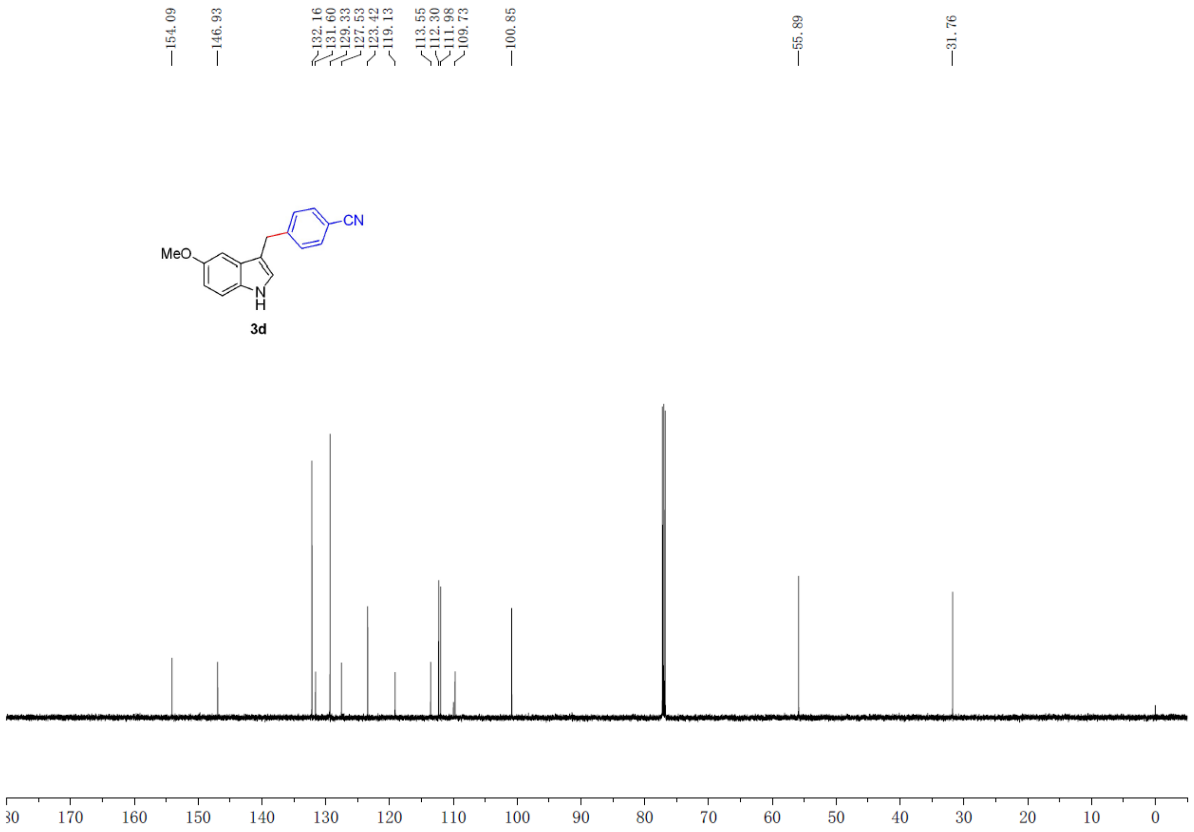

Figure S18: The ^{13}C NMR (151 MHz, $CDCl_3$) of compound **3d**

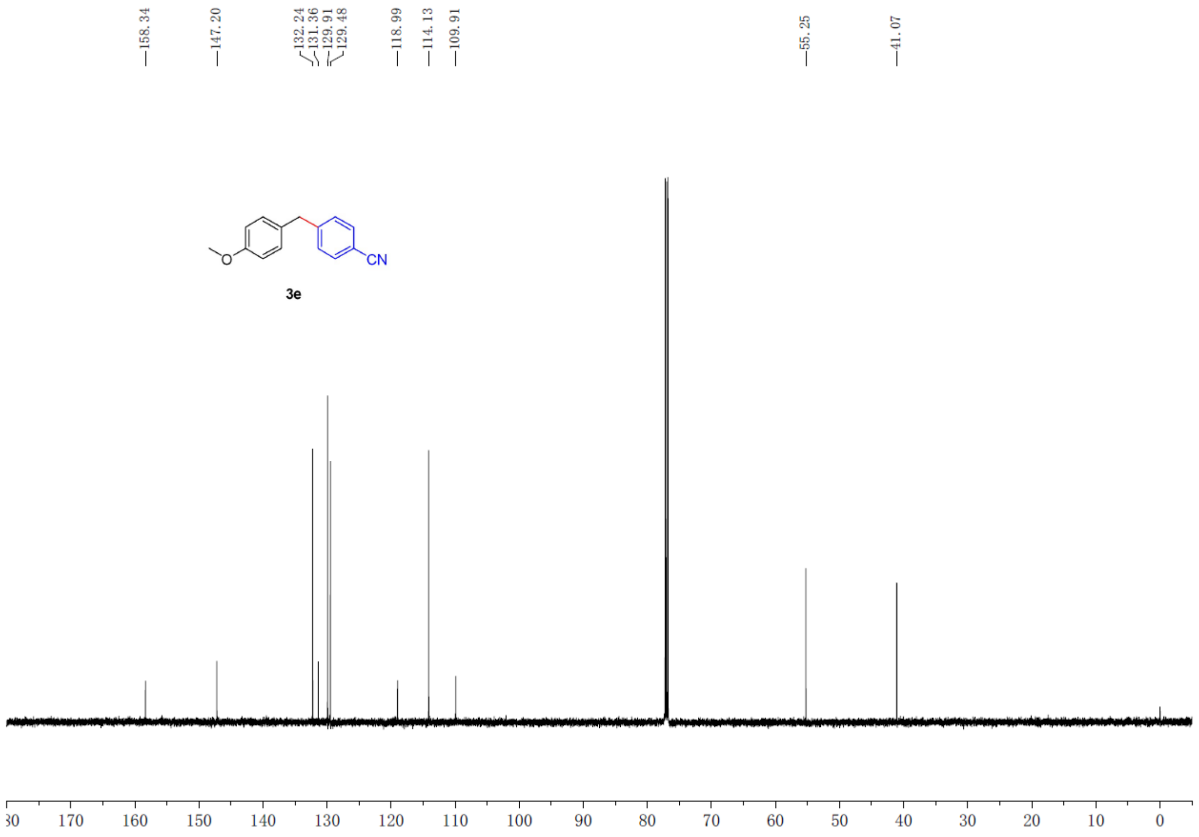

Figure S19: The ^{13}C NMR (151 MHz, $CDCl_3$) of compound **3e**

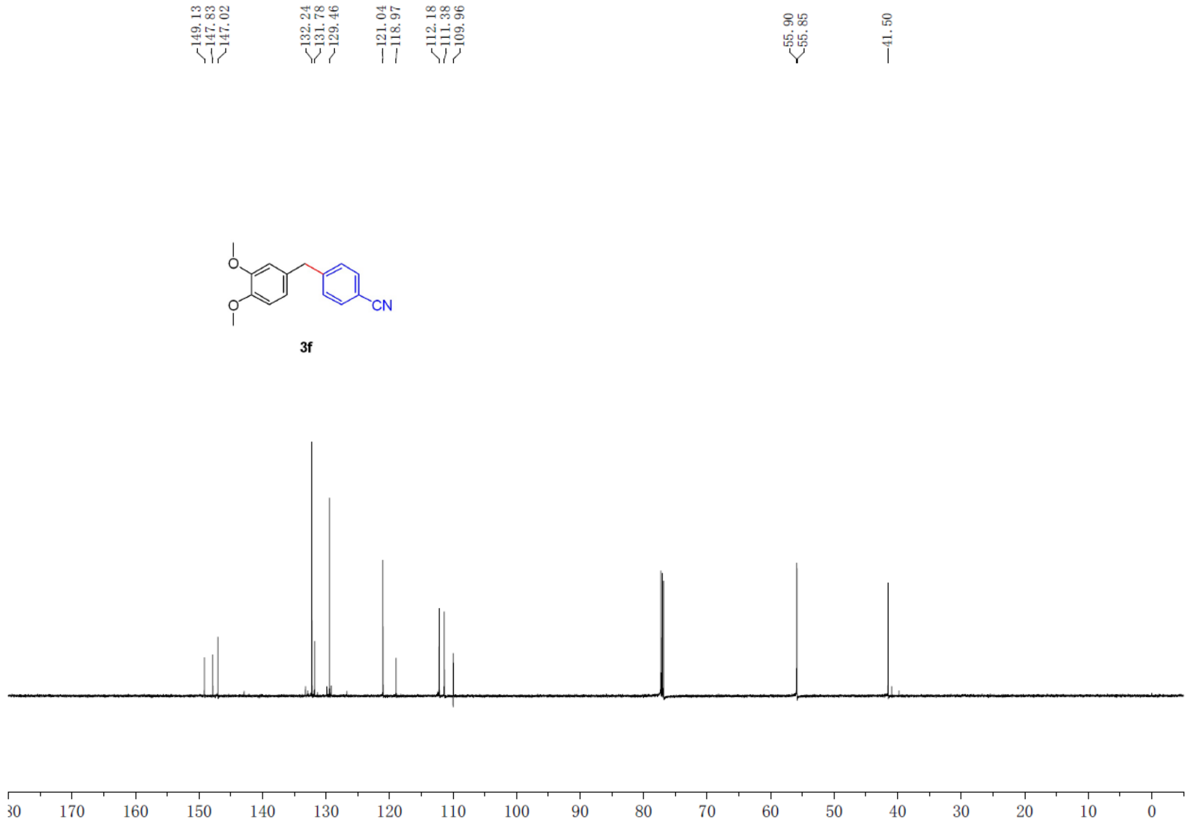

Figure S20: The ^{13}C NMR (151 MHz, $CDCl_3$) of compound **3f**

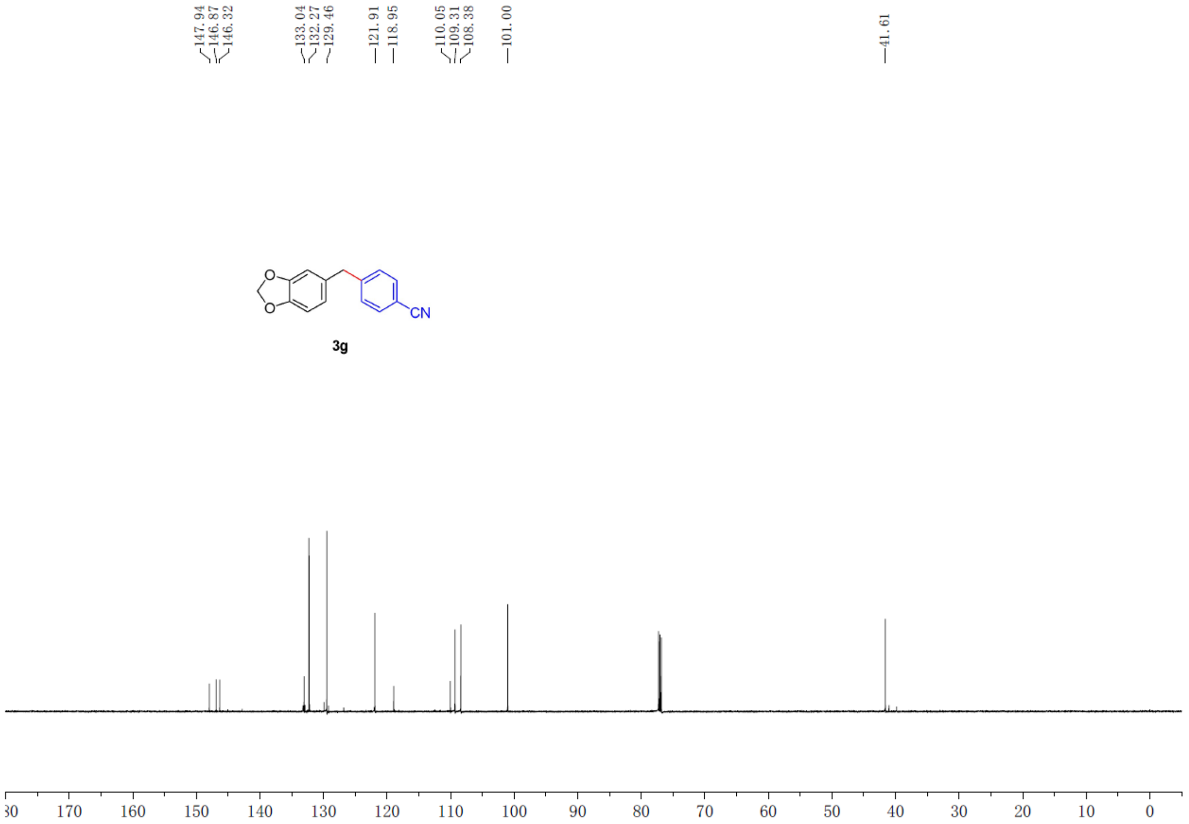

Figure S21: The ^{13}C NMR (151 MHz, $CDCl_3$) of compound **3g**

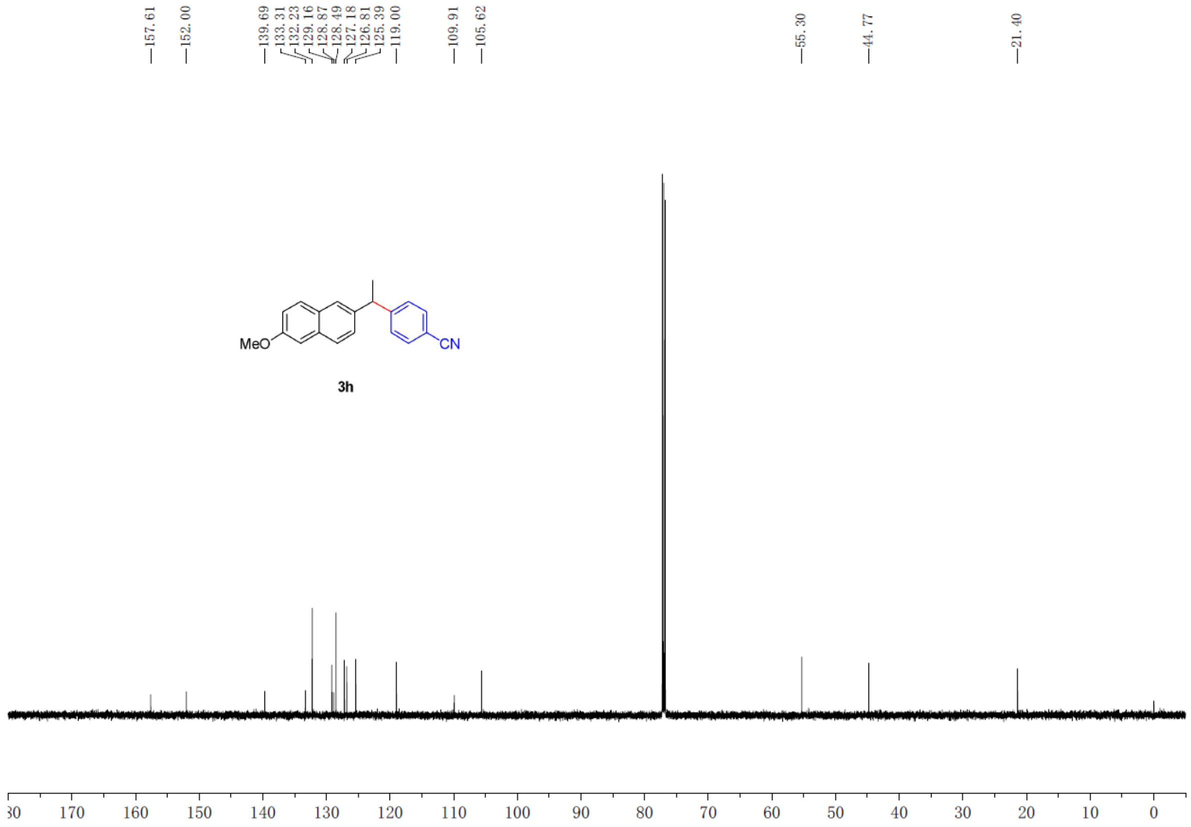

Figure S22: The ^{13}C NMR (151 MHz, $CDCl_3$) of compound **3h**

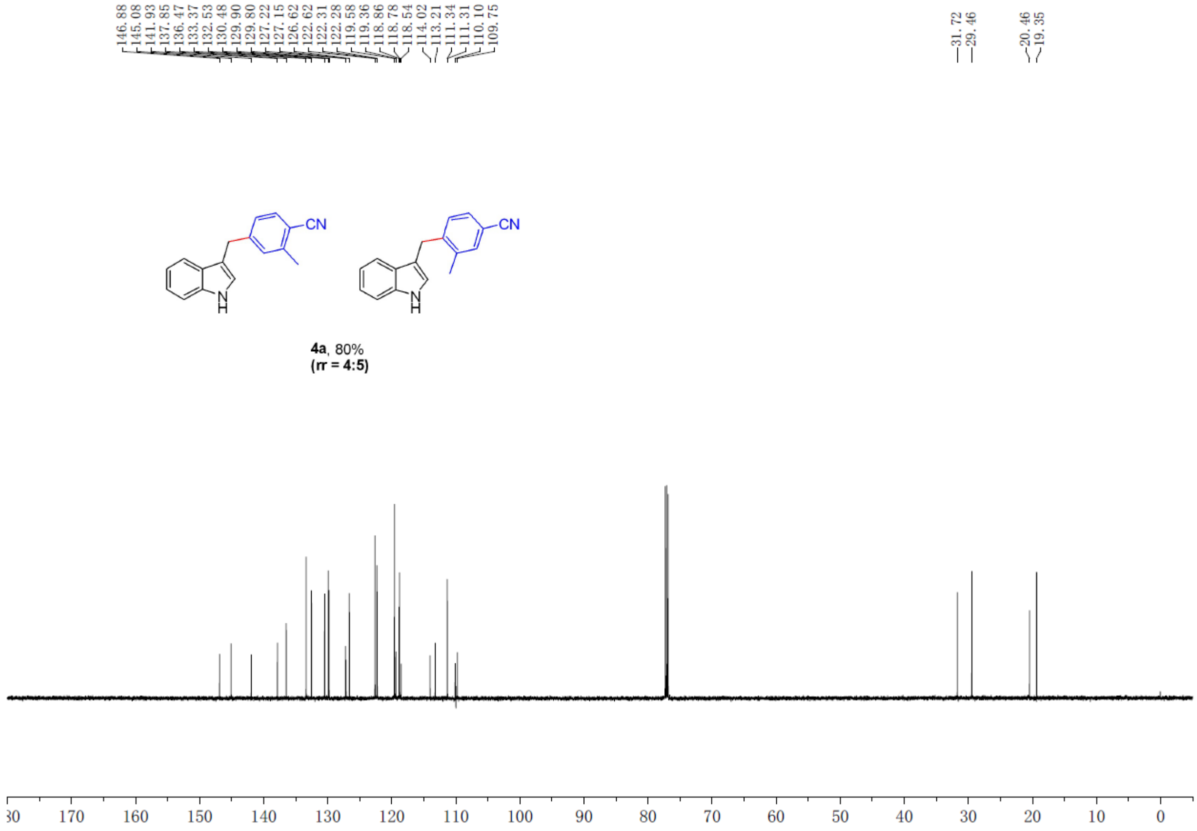

Figure S23: The ^{13}C NMR (151 MHz, $CDCl_3$) of compound **4a**

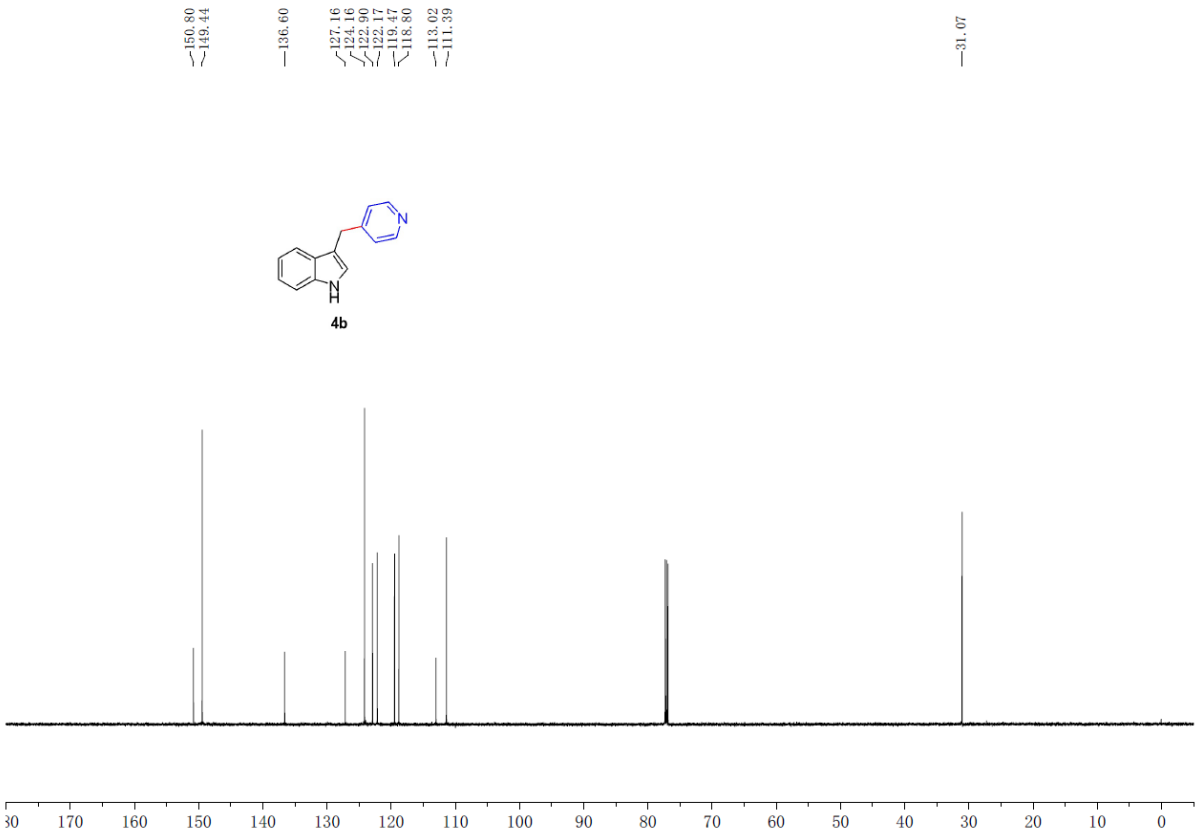

Figure S24: The ^{13}C NMR (151 MHz, $CDCl_3$) of compound **4b**

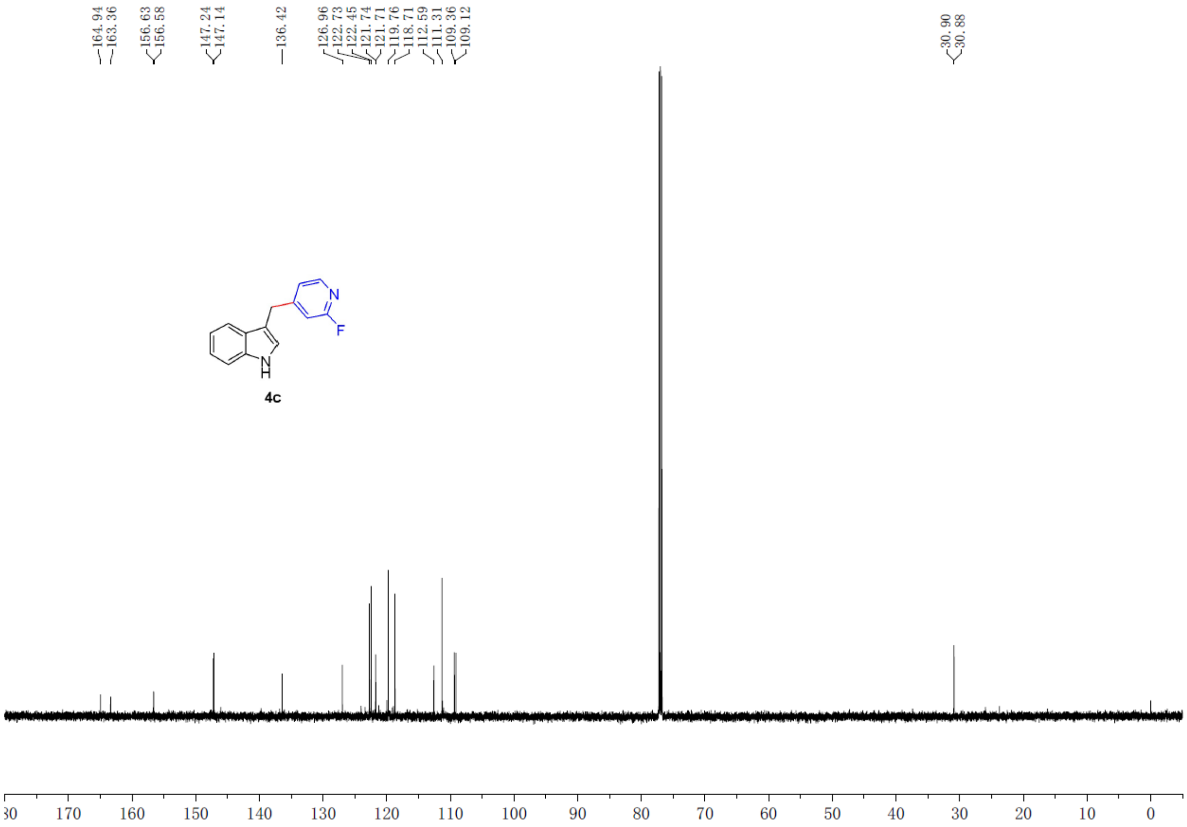

Figure S25: The ^{13}C NMR (151 MHz, $CDCl_3$) of compound **4c**

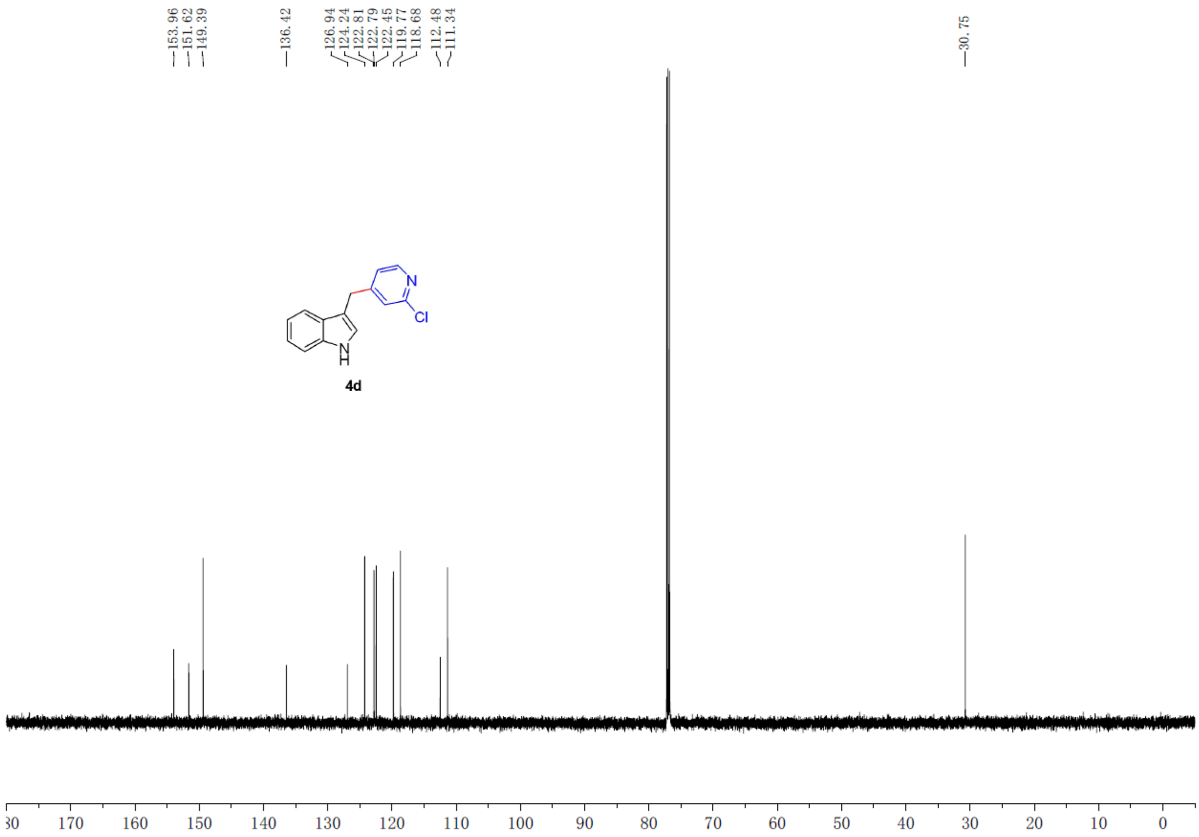

Figure S26: The ^{13}C NMR (151 MHz, $CDCl_3$) of compound **4d**

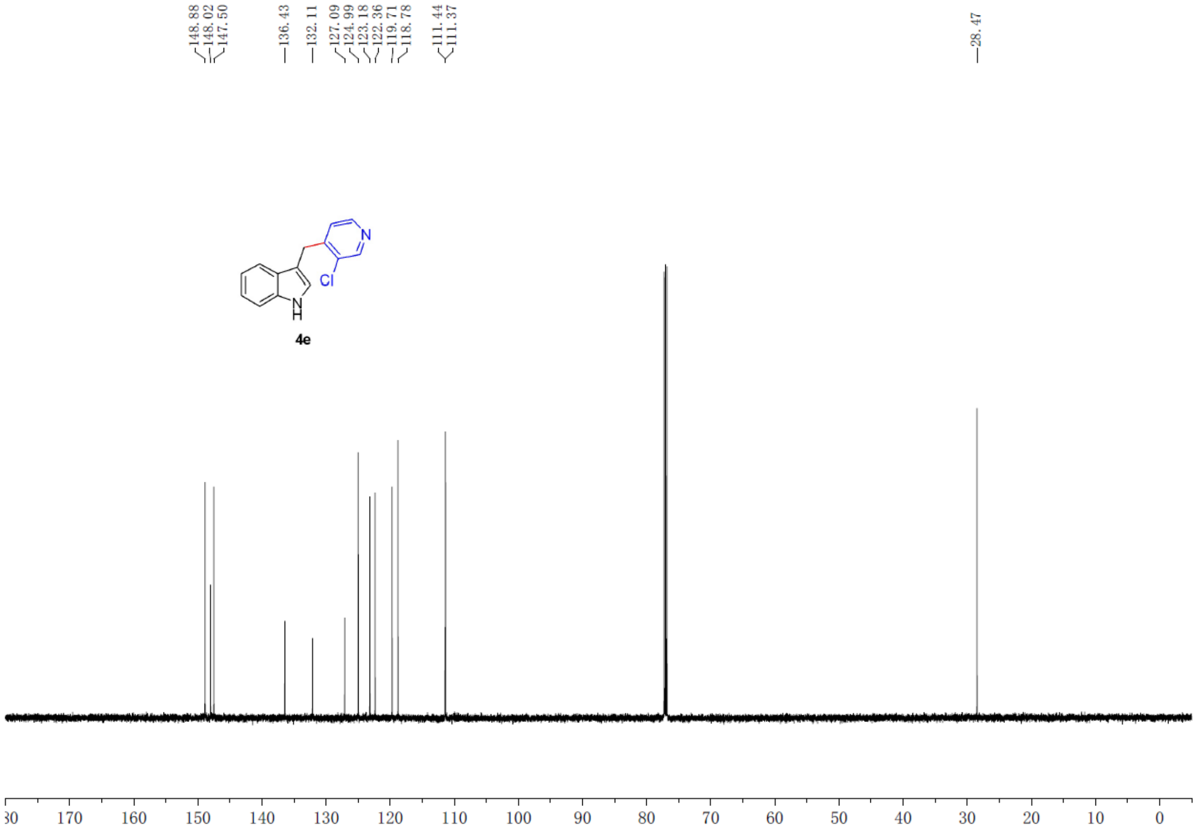

Figure S27: The ^{13}C NMR (151 MHz, $CDCl_3$) of compound **4e**

References

1. Huang, H.; Zhang, G.; Gong, L.; Zhang, S.; Chen, Y. Visible-Light-Induced Chemoselective Deboronative Alkynylation under Biomolecule-Compatible Conditions. *J. Am. Chem. Soc.* **2014,** *136*, 2280–2283.
